# A multi-centre, tolerability study of a cannabidiol-enriched Cannabis Herbal Extract for chronic headaches in adolescents: The CAN-CHA protocol

**DOI:** 10.1371/journal.pone.0290185

**Published:** 2024-09-20

**Authors:** Manik Chhabra, Evan C. Lewis, Robert Balshaw, Breanne Stewart, Zina Zaslawski, Trinity Lowthian, Zahra Alidina, Melila Chesick-Gordis, Wenli Xie, Britt I. Drögemöller, Galen E. B. Wright, Kathryn A. Birnie, Katelynn E. Boerner, Vivian W. L. Tsang, Samantha Lee Irwin, Daniela Pohl, Alexander G. Weil, Erick Sell, Erika Penz, Amy Robson-MacKay, Sophia Mbabaali, Stephanie Blackman, Shanlea Gordon, Jane Alcorn, Richard J. Huntsman, Tim F. Oberlander, G. Allen Finley, Lauren E. Kelly

**Affiliations:** 1 Department of Pharmacology & Therapeutics, Max Rady College of Medicine, University of Manitoba, Winnipeg, Manitoba, Canada; 2 North Toronto Neurology, Toronto, Ontario, Canada; 3 George and Fay Yee Centre for Healthcare Innovation, University of Manitoba, Winnipeg, Manitoba, Canada; 4 Quality Management in Clinical Research (QMCR), University of Alberta, Edmonton, Alberta, Canada; 5 Maternal Infant Child and Youth Research Network (MICYRN), Vancouver, British Columbia, Canada; 6 Youth Research Partners, Childhood Cannabinoid Therapeutics (C4T), Ottawa, Ontario, Canada; 7 Youth Research Partners, Childhood Cannabinoid Therapeutics (C4T), Holland Landing, Ontario, Canada; 8 Youth Research Partners, Childhood Cannabinoid Therapeutics (C4T), Vancouver, British Columbia, Canada; 9 Department of Biochemistry and Medical Genetics, Rady Faculty of Health Sciences, University of Manitoba, Winnipeg, Manitoba, Canada; 10 Department of Anesthesiology, Perioperative and Pain Medicine, University of Calgary, Calgary, Alberta, Canada; 11 Department of Pediatrics, BC Children’s Hospital Research Institute, University of British Columbia, Vancouver, British Columbia, Canada; 12 University of Texas at Austin Pediatric Neurosciences at Dell Children’s Pediatric Headache Program, Austin, Texas, United States of America; 13 Division of Neurology, Children’s Hospital of Eastern Ontario, University of Ottawa, Ottawa, Ontario, Canada; 14 Pediatric Neurosurgery, Department of Surgery, Sainte Justine Hospital, University of Montreal, Montreal, Quebec, Canada; 15 Department of Medicine, College of Medicine, University of Saskatchewan, Saskatoon, Saskatchewan, Canada; 16 Department of Psychiatry, College of Medicine, University of Saskatchewan, Saskatoon, Saskatchewan, Canada; 17 Department of Anesthesia, Pain Management and Perioperative Medicine, Dalhousie University, Halifax, Nova Scotia, Canada; 18 Center for Pediatric Pain Research, IWK Health, Halifax, Nova Scotia, Canada; 19 Cannabinoid Research Initiative of Saskatchewan, University of Saskatchewan, Saskatoon, Saskatchewan, Canada; 20 College of Pharmacy and Nutrition, University of Saskatchewan, Saskatoon, Saskatchewan, Canada; 21 Division Pediatric Neurology, Department of Pediatrics, Faculty of Medicine, Dalhousie University, Halifax, Canada; University of Brescia: Universita degli Studi di Brescia, ITALY

## Abstract

**Introduction:**

Cannabis products have been used in the management of headaches in adults and may play a role in pediatric chronic pain. Canadian pediatricians report increasing use of cannabis for the management of chronic headaches, despite no well-controlled studies to inform its dosing, safety, and effectiveness. The aim of our clinical trial is to determine the dosing and safety of a Cannabidiol (CBD)-enriched Cannabis Herbal Extract (CHE) for the treatment of chronic headaches in adolescents.

**Methods and analysis:**

Youth, parents, and an expert steering committee co-designed this tolerability study. Twenty adolescents (aged 14 to 17 years), with a chronic migraine diagnosis for more than 6 months that has not responded to other therapies will be enrolled into an open label, dose escalation study across three Canadian sites. Study participants will receive escalating doses of a CBD-enriched CHE (MPL-001 with a THC:CBD of 1:25), starting at 0.2–0.4 mg/kg of CBD per day and escalating monthly up to 0.8–1.0 mg/kg of CBD per day. The primary objective of this study is to determine the safety and tolerability of CBD-enriched CHE in adolescents with chronic migraine. Secondary objectives of this study will inform the development of subsequent randomized controlled trials and include investigating the relationship between the dose escalation and change in the frequency of headache, impact and intensity of pain, changes in sleep, mood, function, and quality of life. Exploratory outcomes include investigating steady-state trough plasma levels of bioactive cannabinoids and investigating how pharmacogenetic profiles affect cannabinoid metabolism among adolescents receiving CBD-enriched CHE.

**Discussion:**

This protocol was co-designed with youth and describes a tolerability clinical trial of CBD-enriched CHE in adolescents with chronic headaches that have not responded to conventional therapies. This study is the first clinical trial on cannabis products in adolescents with chronic headaches and will inform the development of future comparative effectiveness clinical trials.

**Trial registration:**

CAN-CHA trial is registered with ClinicalTrials.gov with a number of register NCT05337033.

## Introduction

Globally, chronic headaches are one of the major causes of disability among adolescents. The World Health Organization classifies it under the top ten disabling health conditions [1, 2], with a prevalence of 7.8% in adolescents 14 years of age or older [3–5]. In the US alone, the total annual cost incurred by pediatric headache is estimated around $1.1 billion [6]. Adolescents with chronic headaches often experience reduced quality of life, sleep disruption, anxiety, fatigue, limb pain, dizziness, overuse of medications and academic challenges [7]. Despite advancements in the therapeutic management of chronic pain, treatment of chronic headache disorders in adolescents remains challenging. Non-Steroidal Anti-Inflammatory Drugs (NSAIDs), triptans, gepants, ditans, dopamine antagonists, neuromodulation devices and ergotamine are often used for the acute treatment of migraine [8, 9]. Preventive migraine therapies are limited in availability, efficacy, and authorization for use in the adolescent population. Topiramate is the only FDA approved preventive treatment for migraine in adolescents [1, 10, 11]. In other areas of medicine, cannabinoids have shown therapeutic potential in adolescents where conventional medications fail, including treatment resistant epilepsy, and chemotherapy-induced nausea and vomiting [12]. In adults, use of cannabis products is increasing for the treatment of headaches and migraines [13]. An observational study reported that 36% of adult cannabis users are using cannabis to relieve symptoms related to migraine and/or headaches. Further, this study reported that the use of cannabis products in adults led to an average reduction of 3.6 points on a 10-point intensity of headache scale [14, 15]. Despite promising observational data in adults, a paucity of literature exists that demonstrates the tolerability of cannabis for the treatment of chronic headaches in adolescents. Cannabidiol (CBD) and Tetrahydrocannabinol (THC) are the principal active cannabinoids which have a number of potential therapeutic applications [16]. CBD, which is not associated with the same intoxicating effects of THC, acts as a negative allosteric modulator of CB1 receptors in the endocannabinoid system [17]. CBD potentiates anandamide-mediated intrinsic neurotransmission [18], and has antioxidant and anti-inflammatory activity [19]. Canadian studies have demonstrated the safety and tolerability of CBD-enriched CHE in children with refractory epilepsy [20, 21]. Huntsman et al reported on the preliminary results of the CARE-E trial where a 1:20 THC:CBD CHE oil was found to be well tolerated in children, and THC plasma concentrations were below levels associated with intoxication despite CBD doses of up to 12 mg/kg per day [21].

In Canada, recreational markets have increased cannabis accessibility and there is increasing interest in managing chronic headaches off-label with cannabinoids, self-guided in the absence of evidence [22]. The paucity of clinical trial data in children is due to many historical challenges with studying cannabis products and cannabinoids, resulting from legal difficulties in obtaining products, variable product quality control and the stigma associated with illegal drug use, and particularly with children. The current reality warrants the need to conduct robust interventional studies establishing the tolerability, safety, and efficacy of cannabis in adolescents with chronic headaches. Here we describe a protocol for CAN-CHA (CANnabis for Chronic Headaches in Adolescents) trial, an open-label dose escalation study to establish the tolerability of a CBD-enriched Cannabis Herbal Extract (CHE) in adolescents with chronic headaches.

## Methods and analysis

### Primary objective

To determine the safety and tolerability of escalating doses of a CBD-enriched CHE in adolescents with chronic headaches.

### Secondary objectives

To investigate the relationship between the dose-escalation with headache-free days.

1. To monitor the effect of CBD-enriched CHE oil on the intensity of pain related to chronic headaches.
2. To evaluate the effect of CBD-enriched CHE oil on sleep, mood, and function in adolescents with chronic headaches.
3. To explore the impact of chronic headaches on quality of life.

### Exploratory objectives

1. To investigate the relationship between the dose-escalation and steady-state trough levels of bioactive cannabinoids/endocannabinoids.
2. To study pharmacogenetic variations among adolescents receiving CBD-enriched CHE oil.

### Study population

We will recruit 20 adolescents across three study sites: Halifax, Toronto, and Vancouver. To be eligible to participate in this study, an individual must meet all of the following criteria:

1. Adolescents between 14–17 years of age at the time of screening
2. Diagnosed with Chronic Migraine according to ICHD-3: headache (migraine-like or tension-type like) occurring on 15 or more days per month for more than 3 months, which on at least 8 days per month have features of migraine headache [23].
3. Failed at least two preventive treatment options on the grounds of tolerability and/or efficacy, including but not limited to antidepressants (tricyclic antidepressant or selective norepinephrine reuptake inhibitor), magnesium, gabapentin, topiramate, beta-blockers, memantine, and/or non-pharmacological therapies including nutraceuticals and botox.
4. Females who have reached menarche must have a negative serum pregnancy test during screening
5. Must be willing to engage with psychology and physiotherapy throughout the trial as appropriate.

Adolescents meeting any of the following criteria will be excluded from the study:

1. As per the investigator judgment, the participant is not an ideal candidate due to a personal issue or medical condition that is likely to impede in the successful completion of the study
2. Participants with a history of post-concussion headache or new daily persistent headache
3. Participants with a diagnosis of medication overuse headache
4. Participants with clinically relevant cardiac, renal, or hepatic disease (assessed by the site investigator)
5. Participants with complex regional pain syndrome-II
6. Participants with abnormal ECG findings at baseline (as determined by the investigator)
7. Participants who are on the following medications: opioids, antipsychotics, antimanic, barbiturates, benzodiazepines, muscle relaxants, sedatives, or tramadol
8. Participants with developmental delay or impairments including autism, cerebral palsy, or intellectual disability
9. Participants with a personal or family history of schizophrenia or psychotic disorders
10. Participants who are/plan to become pregnant within the study period or within three months of interventional product discontinuation
11. Participants who cannot commit to using contraception, refraining from recreational cannabis use and driving throughout the study period

### Study design

**Fig 1:**
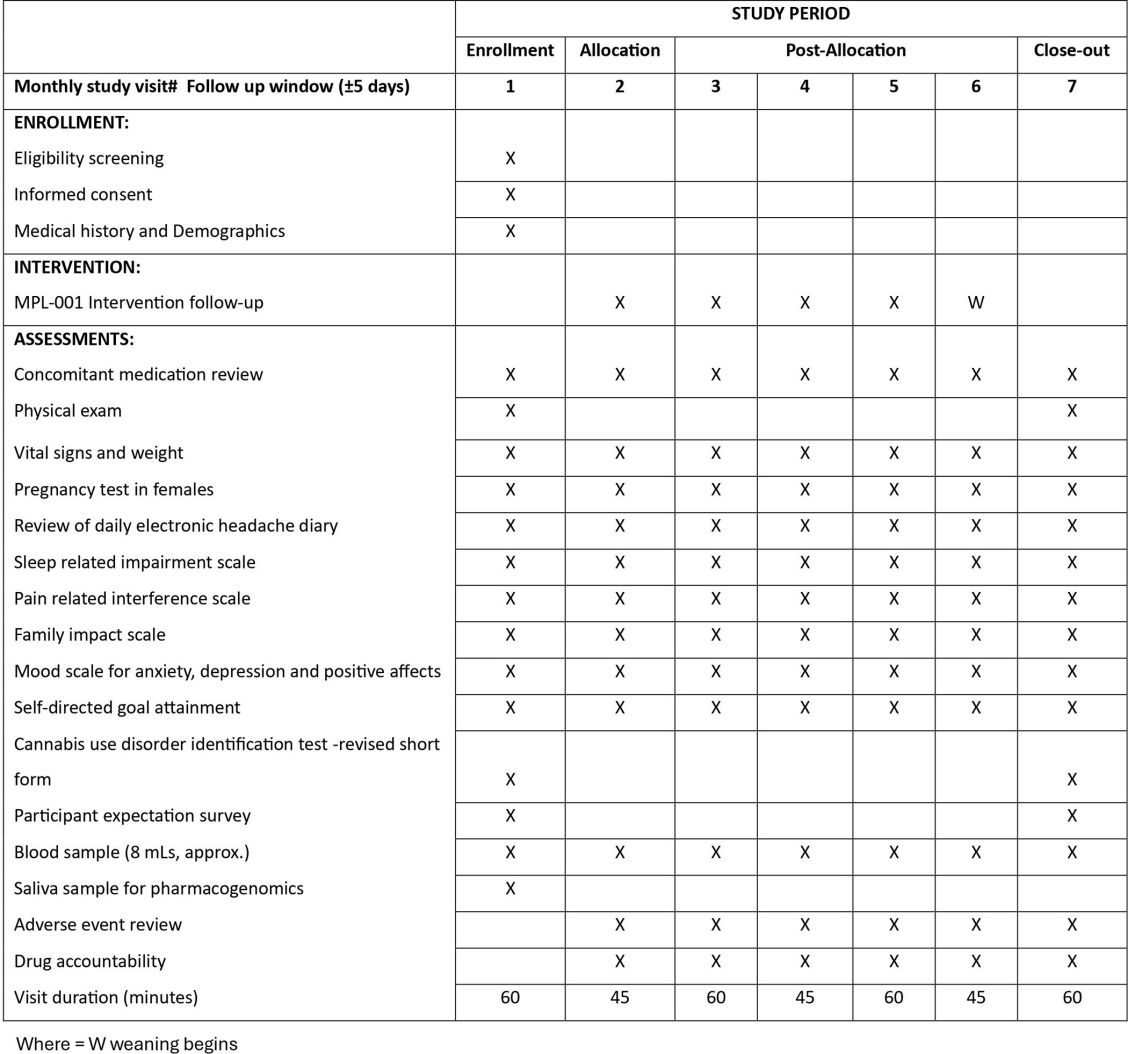
CAN-CHA schedule of events as per SPIRIT guidelines.

CAN-CHA is a multicenter, open-label dose-escalation study to determine the tolerability of MPL-001 in adolescents with treatment refractory chronic migraine. The traditional 3+3 dose-finding design was not practical for our study, primarily due to the intervention’s nature; cannabinoid-based therapies necessitated a ’start low and go slow’ titration strategy. This ensures that all participants would begin with the lowest possible cannabinoid dose followed by a gradual increase in dose. The 3+3 design considers only previous cohort data for determining the next dose assignment, assuming all patients are equivalent and share a similar dose-response relationship. This can introduce bias in identifying the true maximum tolerated dose (MTD) and may lead to suboptimal or potentially harmful dosing. The trial will be conducted in three Canadian centers. CAN-CHA will consist of three different phases: baseline (1 month) without intervention, treatment (4 months of escalating doses) and weaning (1 month). The schedule of events can be found in Fig 1. All participants and their caregivers will be invited to complete a pre- and post-study survey about their experiences in the trial to inform future research.

### Patient and public involvement

CAN-CHA trial was designed in collaboration with youth from the KidsCAN Young Persons’ Research Advisory Group (YRPAG) and the Solutions for Kids in Pain (SKIP) network. The Canadian Collaborative for Childhood Cannabinoid Therapeutics (C4T) Parent Advisory Committee provided insight on the outcome measurement tools and the consent form. Three youth advisors (TL, ZA, MC-G) with chronic migraine have been involved throughout the study design process. They will continue to advise on recruitment strategies, designing materials, reporting and dissemination.

### Intervention

**Fig 2:**
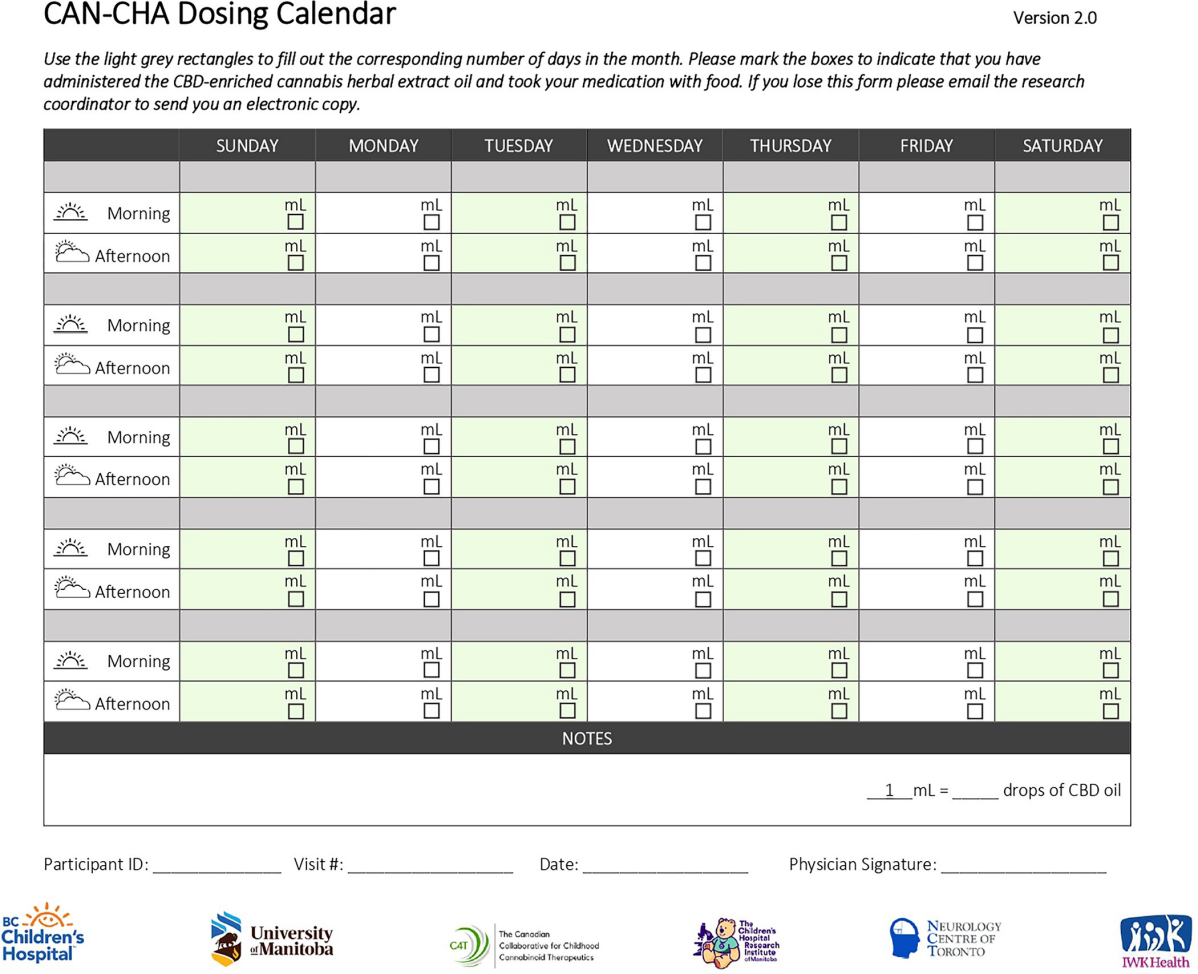
CAN-CHA dosing calendar.

The investigational product for our study is a CBD-enriched CHE, MPL-001, purchased from MediPharm Labs. MPL-001 is a CBD-enriched CHE where each ml of oil contains 2 mg of THC and 50 mg of CBD dissolved in coconut/palm-based medium chain triglycerides (MCT) carrier oil. The MPL-001 (CBD:THC 25:1) oil used in this study contains lemon-peppermint flavoring agents. Manufacturing of MPL-001 occurs following Good Manufacturing Practices includes the following steps: harvesting of plant, followed by weighting, and drying. Further, dried plants are subjected to bucking, ethanolic extraction, filtration of cannabinoid ethanolic solution, and evaporation of ethanol, this leaves over acidic cannabis resin. Subsequently, cannabis resin is decarboxylated (activated) and mixed with oil. The oil is made available in a glass bottle sealed with child lock caps, stored and labeled at the study central pharmacy according in accordance with the *Cannabis Act*, *2018* and the Health Canada Division 5 *Food and Drugs Act* [24]. All the study participants will receive an escalating dose starting at 0.2–0.4 mg/kg of CBD per day with dose increases (0.2 mg/kg/day increments) happening monthly to a maximum of 0.8–1.0 mg/kg of CBD per day. This dosing strategy reflects the current clinical practice of study investigators and well below the maximum dose of CBD-enriched CHE of 10–12 mg/kg/day previously well tolerated in children with epilepsy [21]. Participants will be provided with dosing calendars (Fig 2) and be instructed to take their daily dose bid with 25% of the dose in the morning and 75% of the daily dose in the late afternoon to mimic the diurnal variation [25] in the endocannabinoids and prevents adolescents from having to take cannabis during school hours.

### Dosing rationale and calculations

Data on pharmacokinetics related to cannabinoids in adolescents are extremely lacking. In the current study, the CBD dose is extrapolated from safety data obtained in clinical trials in children and adults with refractory epilepsy [21, 26–33]. CBD was found to be safe and well-tolerated in children with refractory epilepsy at a dose of up to 20 mg/kg/day [26, 27, 29, 32, 34]. We aim to keep the CBD dose as low as possible to limit adverse events and reduce costs for families should we confirm a tolerable dose is effective in future randomized controlled trials. The use of THC is associated with some risk of developing adverse effects of the central nervous system, [35, 36] however, THC possesses its central pain-relieving potential [14, 37]. Previous studies on a 1:20 THC:CBD CHE oil in children reported that plasma THC levels following doses of up to 12 mg/kg/day suggested a low risk for THC intoxication [21]. The quantity of THC in our study product is 1 mg per mL with maximum THC doses only reaching 0.05 mg/kg/day, which is less likely to be associated with any adverse psychoactive reactions with CBD [38, 39]. In this study, the maximum dose of CBD will be less than 10 percent of the recommended dose of CBD in the previous studies conducted in the pediatric population [40]. In order to maintain the accuracy and consistency in the dosing regimen of study participants across all the study centers, the mid-point of the dose range will be selected to calculate the desired dose based upon the weight of the participants. Participants will be weighed at each study visit to assist in tracking changes in appetite; however, the dose calculation will be based on the weight taken at baseline. The final dose will be calculated by rounding off (0.5 mL of MPL-001). This will help in achieving improved precision and will ease administration of the investigational product to the study participants. For example, an adolescent weighing 50 kg will receive a starting total daily dose containing 15 mg of CBD (0.3 mg/kg/day) and take 4 mg in the morning and 11 mg in the afternoon.

### Baseline phase

Eligible adolescents will be asked by their healthcare providers if they are interested in learning more about this research study. A study team member, who is not involved in the patients’ care, will present the study, review the consent documents and answer any questions from the patients and their families. Adolescents who meet the inclusion criteria, assent, and whose caregiver’s consent to participate will then begin a baseline period. Baseline will consist of one month period without intervention to estimate headache frequency and severity, as well as mood, sleep, and pain before administering the intervention to the participants. Participants will be asked to maintain a daily electronic headache diary that will include reporting on the severity of headaches, associated pain, sleep, absences from work/school and adverse events. Participants will complete age-validated scales on sleep related impairment, anxiety, depression, positive mood, pain interference, family impact and goal attainment scaling [41–46] as described in the Fig 1. A blood sample will be drawn from the study participants to measure liver transaminases (ALT/AST) and creatinine, measure endocannabinoids and detect pregnancies, while saliva will be sampled for extraction of genomic DNA to allow for genotyping of pharmacogenetic variants. ECGs (within 3 months of screening or done at the time of screening) will be recorded at baseline to ensure there are no cardiac electrical activity concerns.

### Treatment phase

Following the one-month baseline period, study participants will receive CBD-enriched CHE oil, MPL-001with a dosing calendar and administration pamphlet shipped directly to their homes from the trial coordinating centre. A handout with video component on cannabis oil administration co-created with the youth advisors will be provided to all study participants and their families. There will also be a demonstration by the research coordinator at the first study visit using olive oil in a product bottle. The study participants will be instructed to administer the investigational product at a starting dose of 0.2–0.4 mg/kg/day divided into two doses (BID, 25% in the morning and 75% in the evening after school) each day for one month. Dose-escalation schedule during the treatment phase is described in Table 1. Participants will continue to complete a daily electronic headache diary to monitor symptoms and adverse effects. During all follow up visits, participants and their caregivers will be asked to complete validated outcome measurement tools alongside the PedsQL ™ Family Impact Module assessment, and Self-directed goal attainment. Blood samples will be collected prior to starting the next dose level, at study visits and will be used to evaluate changes and variability in cannabinoid pharmacokinetics with escalating doses, confirm pregnancies, and monitor liver enzymes and creatinine over time.

**Table 1:** Dosing schedule for CAN-CHA evaluating a CBD-enriched CHE from visit 2 to visit 6.

Visit	CBD total daily dose	Frequency	Duration
Visit 2	0.2–0.4 mg/kg/day	BID	1 month
Visit 3	0.4–0.6 mg/kg/day	BID	1 month
Visit 4	0.6–0.8 mg/kg/day	BID	1 month
Visit 5	0.8–1 mg/kg/day	BID	1 month

### Weaning phase

After the baseline phase (one month, no treatment) and the treatment phase (four months, escalating doses), participants will start the weaning schedule. Weaning includes incrementally reducing the dose (by 0.2 mg/kg CBD every week) leading to complete discontinuation of the study product. The complete weaning schedule is detailed in Table 2. The intervention will be discontinued by visit 7. If the parents, adolescents, and healthcare providers feel that there was improvement while on the intervention, participants will discuss the continued authorization of medical cannabis with the enrolling physician, caregivers, and their healthcare team.

**Table 2:** The weaning schedule which begins at visit 6 and ends at visit 7.

Visit 6	CBD total daily dose	Frequency	Duration
Week 1	0.6–0.8 mg/kg/day	BID	7 days
Week 2	0.4–0.6 mg/kg/day	BID	7 days
Week 3	0.2–0.4 mg/kg/day	BID	7 days
Week 4	0 mg/kg/day	BID	7 days

### Withdrawal criteria

Study participants may withdraw from the study at any point. If a participant’s headaches worsen or they suffer from intolerable treatment related adverse effects, they will be withdrawn from the study. Participants who become pregnant during the study period, do not attend follow-up visits, or do not comply with the prescribed interventional drug regimen will be withdrawn from the study. Participants will be given the option to only withdraw from the study intervention, these participants will continue to be followed up for safety assessments till the end of study. All participants withdrawn from the study will be included in the final report at the end of the study for transparency.

### Dose limiting toxicities (DLTs)

Adverse events will be categorized using the Common Terminology Criteria for Adverse Events (CTCAE version 5.0 dated 27 Nov 2017). If any of the following DLTs occur the participant will not move up to the next dose level. Dose escalations will not be reattempted but the participant shall remain in CAN-CHA should no other DLTs occur. DLTs include:

1. Parental/youth report complaints of moderate mood elevation defined as exaggerated feelings of well-being which is disproportionate to the events and stimuli (Euphoria Grade 2)
2. Somnolence Grade 2 which includes moderate sedation (sleepiness and drowsiness) that limits instrumental activities of daily living
3. Cannabis-attributed diarrhea, Grade 2 or more defined as an increase of 4–6 stools per day over baseline; moderate increase in ostomy output compared to baseline; limiting instrumental activities of daily living
4. Unexplained tachycardia (w/out pain, fever, anemia etc.) requiring medical intervention
5. Unexplained hypotension requiring medical intervention
6. Non-infectious conjunctivitis Grade 2 defined as moderate decrease in visual acuity (best corrected visual acuity 20/40 and better or 3 lines or less decreased vision from known baseline) characterized by inflammation, swelling and redness to the conjunctiva of the eye.
7. Serious adverse events requiring hospitalization
8. Discretion of the participant, physician, or parents

### Primary outcome

The frequency and type of cannabis-related adverse events among study participants will be assessed daily throughout the study. Adverse events will be reported daily and reviewed at study visits.

### Secondary outcomes

1. The frequency of headache measured using headache-free days [assessed: daily throughout the study]. Reported number of headache-free days per month during the study period
2. The average intensity of pain due to chronic headache as measured using an 11-point Numeric Rating Scale (NRS) [assessed: daily throughout the study] [47]. Reported for each study participant as a percentage change in average daily pain intensity due to chronic headaches on the numeric rating scale (NRS) from baseline to each follow up visit
3. The impact of pain on participants’ quality of life using the PROMIS Pediatric Pain Interference–Short Form 8a [assessed: at visits 1,2,3,4,5,6 and 7] [41]. Reported as a percentage change in the scores from baseline value
4. The quality of sleep will be recorded using the PROMIS Pediatric Sleep-Related Impairment–Short Form 8a scale [48]. [assessed: at visits 1,2,3,4,5,6 and 7] Reported as a percentage change in scores from baseline value
5. Changes in anxiety will be measured using the PROMIS Pediatric Short Form v2.0—Anxiety - 8a scale [49] [assessed: at visits 1,2,3,4,5,6 and 7]. Reported as percentage change in scores from the baseline value
6. Change in mood will be evaluated using two tools PROMIS Pediatric Short Form v2.0—Depressive Symptoms 8a scale and the PROMIS Pediatric Positive Affect–Short Form 8a. [44, 50] [assessed: at visits 1,2,3,4,5,6 and 7]. Reported as a percentage change in scores from baseline value
7. Change in self-directed goal attainment (participant and parent reported) [assessed: monthly throughout the study] [45]. Reported as a percentage toward a physical, mental and social by participant at each monthly visit
8. Change in scores of PedsQL ™ Family Impact Module, Version 2.0 [assessed: monthly throughout the study] [46]. Reported as percentage change in scores from the baseline value
9. Steady-state trough plasma levels of bioactive cannabinoids THC, CBD, 11-OH-THC, 7-OH-CBD, and endocannabinoids [assessed: monthly throughout the study]. Reported as a plasma concentration relative to each dose increase/decrease and according to genotype
10. Genetic polymorphisms within genes encoding for cytochrome P450 enzymes and the p-glycoprotein transporter and their association with plasma levels of THC, CBD, and their active metabolites in the study participants

### Sample size

CAN-CHA is a tolerability study, designed to evaluate the safety of escalating doses of a cannabidiol-enriched CHE. Given the within-participant study design, a sample size of 20 study participants (common for early phase trials) should provide a reasonable characterization of the pattern of adverse event frequency and severity as dosing increases. To provide more generalizable data we will recruit these participants across three pediatric chronic pain/headache programs in Halifax, Vancouver, and Toronto, Canada.

### Data collection, management and sharing

Data collection will be the responsibility of the study team at each site under the supervision of the site investigators (TFO, ECL, GAF) and trial sponsor (LEK, University of Manitoba). Investigators and research coordinators will be responsible for ensuring the accuracy, completeness, legibility, and timeliness of the data reported. All source documents will be completed in a neat, legible manner to ensure accurate interpretation of data. Hardcopies of the study visit measurement tool will be provided as source documents for each participant enrolled in the study. Data recorded in the electronic case report form (eCRF) derived from source documents should be consistent with source documents. Clinical and laboratory data will be entered into REDCap (Research Electronic Data Capture), [51] a 21 CFR Part 11-compliant data capture system provided by the Women and Children’s Health Research Institute at the University of Alberta, Edmonton. REDCap includes password protection and internal data quality checks, such as automatic range checking, to identify data that appear inconsistent, incomplete, or inaccurate to the study team for verification. Clinical data will be entered directly from the source documents. Full de-identified datasets will be available from the corresponding author upon reasonable request and review by the trial steering committee.

### Statistical analysis

The primary statistical analyses will be a descriptive summary of the pattern of incidence and severity of adverse events across dosage levels. Conventional summary statistics will be used to describe baseline characteristics and other outcomes (means, standard deviations, as well as medians, range, and interquartile range (IQR) for numerical variables; counts and percentages for categorical variables). Adverse events will be reported overall (duration of the study period) and by dosage level (study month). Medians, ranges and IQR will be provided for the concentrations of CBD, THC, and the major metabolites at each sampling point. The ratio of concentration of parent compound to metabolites and endocannabinoids will also be summarized to explore variability in cannabinoid metabolism. Severity, frequency, and relationship of treatment emergent AEs to study intervention will be presented by system organ class and MedDRA codes. The secondary outcomes including pain, sleep impairment, depression, positive affect, anxiety, and goal attainment scores will be summarized at each timepoint; within-participant change from baseline of these measures will also be summarized (both absolute and percentage change) but this trial is underpowered, and not designed to evaluate efficacy.

### Ethics and dissemination

CAN-CHA received a No Objection Letter from Health Canada (Dec 2022), registered with ClinicalTrials.gov (NCT05337033). The study was approved by the University of Manitoba Health Research Ethics Board (HS25503- B2022:037). An institutional cannabis research license was received in Sep 2022, and we expect to enroll our first participant in 2024. Written informed consent will be received from all the participants and from the legal guardians for the participants who will be below 16 years of age. We plan to disseminate our findings of the CAN-CHA trial by presenting them to conferences, sharing them with participants and the public using infographics, and publishing the results in an open-access journal.

## Discussion

Adolescents with refractory headache disorders are using cannabis products off-label to manage their symptoms, self-guided in the absence of evidence [52–54]. Chronic headaches are often resistant to standard drug therapies in adolescents, resulting in school absenteeism, withdrawal from social activities and can cause significant stress for families [55]. Based on anecdotal reports, parents of children with chronic headaches found cannabinoids to be effective for the management of headache [1, 56], but there remains minimal data available to inform dosing or safety. CAN-CHA is a tolerability study designed with youth and parents that will investigate the safety of escalating doses up to 1 mg/kg/day of CBD-enriched CHE in adolescents with chronic headache. This represents the first study evaluating a cannabis product, providing valuable knowledge on the safety and dosing of a CBD-enriched CHE in children with chronic pain. The CAN-CHA trial is an open label with a small sample size, underpowered to evaluate efficacy. Additionally, due to smaller sample size it is not feasible to conduct a dose response modelling due to increased variability, challenging to generalize findings to larger population and reduced statistical power [57]. This study will inform dose selection for a larger scale randomized clinical trial comparing a CBD-enriched CHE oil to placebo (on top of standard of care) for adolescents with chronic headache. In this trial, we are using CBD-enriched CHE with low THC due to the reported protective effect of CBD on THC toxicity, as documented in studies conducted in the late 19s [38, 39]. The potential risks and unknowns regarding the impact of cannabinoids on the developing brain are clearly communicated in the consent and assent forms. In addition to this clinical trial, we are committed to gathering long-term real-world observational evidence to enhance our understanding of how cannabinoids affects children’s health and quality of life for families (https://classic.clinicaltrials.gov/ct2/show/NCT05863910)). Given the high prevalence of chronic headaches, severe morbidity, and current lack of effective treatment options, the successful outcomes from this research project will have the potential to create meaningful impact for the lives of Canadian adolescents.

## Supporting information

S1 Checklist Recommended items to address in a clinical trial protocol and related documents. (PDF)

S1 Data Study protocol reviewed by ethics committee. (PDF)

S2 Data Cannabinoids and metabolite assessment. (PDF)

S1 File (DOCX)

## Abbreviations

AE: Adverse Events
BMI: Body Mass Index
CBD: Cannabidiol
CHE: Cannabis Herbal Extract
CNS: Central Nervous System
eCRF: electronic case report form
GMP: Good Manufacturing Practices
NRS: Numeric Rating Scale
NSAIDs: Non-steroidal Anti-inflammatory Drugs
REDCap: Research Electronic Data Capture
THC: delta 9 –Tetrahydrocannabinol

## References

[1] Seshia SS, Wang SJ, Abu-Arafeh I, Hershey AD, Guidetti V, Winner P, et al. Chronic daily headache in children and adolescents: a multi-faceted syndrome. Can J Neurol Sci. 2010;37(6):769–78. Epub 2010/11/10. doi: 10.1017/s0317167100051428 . 21059537

[2] Stovner L, Hagen K, Jensen R, Katsarava Z, Lipton R, Scher A, et al. The global burden of headache: a documentation of headache prevalence and disability worldwide. Cephalalgia. 2007;27(3):193–210. Epub 2007/03/27. doi: 10.1111/j.1468-2982.2007.01288.x . 17381554

[3] Castillo J, Muñoz P, Guitera V, Pascual J. Kaplan Award 1998. Epidemiology of chronic daily headache in the general population. Headache. 1999;39(3):190–6. Epub 2004/12/23. doi: 10.1046/j.1526-4610.1999.3903190.x . 15613213

[4] Lantéri-Minet M, Auray JP, El Hasnaoui A, Dartigues JF, Duru G, Henry P, et al. Prevalence and description of chronic daily headache in the general population in France. Pain. 2003;102(1–2):143–9. Epub 2003/03/07. doi: 10.1016/s0304-3959(02)00348-2 . 12620605

[5] Grande RB, Aaseth K, Gulbrandsen P, Lundqvist C, Russell MB. Prevalence of primary chronic headache in a population-based sample of 30- to 44-year-old persons. The Akershus study of chronic headache. Neuroepidemiology. 2008;30(2):76–83. Epub 2008/02/16. doi: 10.1159/000116244 . 18277081

[6] Law EF, Palermo TM, Zhou C, Groenewald CB. Economic Impact of Headache and Psychiatric Comorbidities on Healthcare Expenditures Among Children in the United States: A Retrospective Cross-Sectional Study. Headache. 2019;59(9):1504–15. Epub 2019/09/15. doi: 10.1111/head.13639 ; PubMed Central PMCID: PMC6818708. 31520418 PMC6818708

[7] Bellini B, Arruda M, Cescut A, Saulle C, Persico A, Carotenuto M, et al. Headache and comorbidity in children and adolescents. J Headache Pain. 2013;14(1):79. Epub 2013/09/26. doi: 10.1186/1129-2377-14-79 ; PubMed Central PMCID: PMC3849985. 24063537 PMC3849985

[8] Pardutz A, Schoenen J. NSAIDs in the Acute Treatment of Migraine: A Review of Clinical and Experimental Data. Pharmaceuticals (Basel). 2010;3(6):1966–87. Epub 2010/06/17. doi: 10.3390/ph3061966 ; PubMed Central PMCID: PMC4033962. 27713337 PMC4033962

[9] Ravishankar K, Chakravarty A, Chowdhury D, Shukla R, Singh S. Guidelines on the diagnosis and the current management of headache and related disorders. Ann Indian Acad Neurol. 2011;14(Suppl 1):S40–59. Epub 2011/08/19. doi: 10.4103/0972-2327.83100 ; PubMed Central PMCID: PMC3152170. 21847329 PMC3152170

[10] Oermann CM, Retsch-bogart GZ, Quittner AL, Gibson RL, McCoy KS, Montgomery AB, et al. An 18-month study of the safety and efficacy of repeated courses of inhaled aztreonam lysine in cystic fibrosis. Pediatric Pulmonology. 2010;45(11):1121–34. doi: 10.1002/ppul.21301 20672296 PMC3867945

[11] Kacperski J, Kabbouche MA, O’Brien HL, Weberding JL. The optimal management of headaches in children and adolescents. Ther Adv Neurol Disord. 2016;9(1):53–68. Epub 2016/01/21. doi: 10.1177/1756285615616586 ; PubMed Central PMCID: PMC4710107. 26788131 PMC4710107

[12] Lochte BC, Beletsky A, Samuel NK, Grant I. The Use of Cannabis for Headache Disorders. Cannabis Cannabinoid Res. 2017;2(1):61–71. Epub 2017/09/02. doi: 10.1089/can.2016.0033 ; PubMed Central PMCID: PMC5436334. 28861505 PMC5436334

[13] Salazar CA, Tomko RL, Akbar SA, Squeglia LM, McClure EA. Medical Cannabis Use among Adults in the Southeastern United States. Cannabis. 2019;2(1):53–65. Epub 2019/03/01. doi: 10.1016/j.drugalcdep.2010.02.017 ; PubMed Central PMCID: PMC6388700. 30815638 PMC6388700

[14] Rhyne DN, Anderson SL, Gedde M, Borgelt LM. Effects of Medical Marijuana on Migraine Headache Frequency in an Adult Population. Pharmacotherapy. 2016;36(5):505–10. Epub 2016/01/11. doi: 10.1002/phar.1673 . 26749285

[15] Cuttler C, Spradlin A, Cleveland MJ, Craft RM. Short- and Long-Term Effects of Cannabis on Headache and Migraine. J Pain. 2020;21(5–6):722–30. Epub 2019/11/13. doi: 10.1016/j.jpain.2019.11.001 . 31715263

[16] Gonçalves J, Rosado T, Soares S, Simão AY, Caramelo D, Luís Â, et al. Cannabis and Its Secondary Metabolites: Their Use as Therapeutic Drugs, Toxicological Aspects, and Analytical Determination. Medicines (Basel). 2019;6(1). Epub 2019/03/01. doi: 10.3390/medicines6010031 ; PubMed Central PMCID: PMC6473697. 30813390 PMC6473697

[17] Morales P, Goya P, Jagerovic N, Hernandez-Folgado L. Allosteric Modulators of the CB(1) Cannabinoid Receptor: A Structural Update Review. Cannabis Cannabinoid Res. 2016;1(1):22–30. Epub 2016/01/01. doi: 10.1089/can.2015.0005 ; PubMed Central PMCID: PMC5576597. 28861476 PMC5576597

[18] Pertwee RG. Pharmacology of cannabinoid receptor ligands. Curr Med Chem. 1999;6(8):635–64. Epub 1999/09/02. . 10469884

[19] Benito C, Tolón RM, Pazos MR, Núñez E, Castillo AI, Romero J. Cannabinoid CB2 receptors in human brain inflammation. Br J Pharmacol. 2008;153(2):277–85. Epub 2007/10/16. doi: 10.1038/sj.bjp.0707505 ; PubMed Central PMCID: PMC2219537. 17934510 PMC2219537

[20] Reithmeier D, Tang-Wai R, Seifert B, Lyon AW, Alcorn J, Acton B, et al. The protocol for the Cannabidiol in children with refractory epileptic encephalopathy (CARE-E) study: a phase 1 dosage escalation study. BMC Pediatr. 2018;18(1):221. Epub 2018/07/10. doi: 10.1186/s12887-018-1191-y ; PubMed Central PMCID: PMC6035794. 29981580 PMC6035794

[21] Huntsman RJ, Tang-Wai R, Alcorn J, Vuong S, Acton B, Corley S, et al. Dosage Related Efficacy and Tolerability of Cannabidiol in Children With Treatment-Resistant Epileptic Encephalopathy: Preliminary Results of the CARE-E Study. Front Neurol. 2019;10:716. Epub 2019/07/25. doi: 10.3389/fneur.2019.00716 ; PubMed Central PMCID: PMC6616248. 31333569 PMC6616248

[22] Rieder MJ. Is the medical use of cannabis a therapeutic option for children? Paediatr Child Health. 2016;21(1):31–4. Epub 2016/03/05. doi: 10.1093/pch/21.1.31 ; PubMed Central PMCID: PMC4758425. 26941559 PMC4758425

[23] Headache Classification Committee of the International Headache Society (IHS) The International Classification of Headache Disorders, 3rd edition. Cephalalgia. 2018;38(1):1–211. Epub 2018/01/26. doi: 10.1177/0333102417738202 . 29368949

[24] Rotermann M. Looking back from 2020, how cannabis use and related behaviours changed in Canada. Health Rep. 2021;32(4):3–14. Epub 2021/04/22. doi: 10.25318/82-003-x202100400001-eng . 33881274

[25] Vaughn LK, Denning G, Stuhr KL, de Wit H, Hill MN, Hillard CJ. Endocannabinoid signalling: has it got rhythm? British journal of pharmacology. 2010;160(3):530–43. doi: 10.1111/j.1476-5381.2010.00790.x 20590563 PMC2931554

[26] Devinsky O, Patel AD, Thiele EA, Wong MH, Appleton R, Harden CL, et al. Randomized, dose-ranging safety trial of cannabidiol in Dravet syndrome. Neurology. 2018;90(14):e1204–e11. Epub 2018/03/16. doi: 10.1212/WNL.0000000000005254 ; PubMed Central PMCID: PMC5890607. 29540584 PMC5890607

[27] Devinsky O, Patel AD, Cross JH, Villanueva V, Wirrell EC, Privitera M, et al. Effect of Cannabidiol on Drop Seizures in the Lennox-Gastaut Syndrome. N Engl J Med. 2018;378(20):1888–97. Epub 2018/05/17. doi: 10.1056/NEJMoa1714631 . 29768152

[28] Thiele E, Marsh E, Mazurkiewicz-Beldzinska M, Halford JJ, Gunning B, Devinsky O, et al. Cannabidiol in patients with Lennox-Gastaut syndrome: Interim analysis of an open-label extension study. Epilepsia. 2019;60(3):419–28. Epub 2019/02/12. doi: 10.1111/epi.14670 ; PubMed Central PMCID: PMC6850399. 30740695 PMC6850399

[29] Devinsky O, Marsh E, Friedman D, Thiele E, Laux L, Sullivan J, et al. Cannabidiol in patients with treatment-resistant epilepsy: an open-label interventional trial. Lancet Neurol. 2016;15(3):270–8. Epub 2016/01/03. doi: 10.1016/S1474-4422(15)00379-8 . 26724101

[30] Raucci U, Pietrafusa N, Paolino MC, Di Nardo G, Villa MP, Pavone P, et al. Cannabidiol Treatment for Refractory Epilepsies in Pediatrics. Front Pharmacol. 2020;11:586110. Epub 2020/10/30. doi: 10.3389/fphar.2020.586110 ; PubMed Central PMCID: PMC7550750. 33117180 PMC7550750

[31] Abu-Sawwa R, Scutt B, Park Y. Emerging Use of Epidiolex (Cannabidiol) in Epilepsy. J Pediatr Pharmacol Ther. 2020;25(6):485–99. Epub 2020/08/26. doi: 10.5863/1551-6776-25.6.485 ; PubMed Central PMCID: PMC7439947. 32839652 PMC7439947

[32] Devinsky O, Nabbout R, Miller I, Laux L, Zolnowska M, Wright S, et al. Long-term cannabidiol treatment in patients with Dravet syndrome: An open-label extension trial. Epilepsia. 2019;60(2):294–302. Epub 2018/12/26. doi: 10.1111/epi.14628 ; PubMed Central PMCID: PMC7379690. 30582156 PMC7379690

[33] Huntsman RJ, Kelly LE, Alcorn J, Appendino JP, Bélanger RE, Crooks B, et al. Improving the regulation of medical cannabis in Canada to better serve pediatric patients. Cmaj. 2021;193(41):E1596–e9. Epub 2021/10/20. doi: 10.1503/cmaj.202169 ; PubMed Central PMCID: PMC8547246 34663604 PMC8547246

[34] Miller I, Scheffer IE, Gunning B, Sanchez-Carpintero R, Gil-Nagel A, Perry MS, et al. Dose-Ranging Effect of Adjunctive Oral Cannabidiol vs Placebo on Convulsive Seizure Frequency in Dravet Syndrome: A Randomized Clinical Trial. JAMA Neurol. 2020;77(5):613–21. Epub 2020/03/03. doi: 10.1001/jamaneurol.2020.0073 ; 32119035 PMC7052786

[35] Rubino T, Realini N, Braida D, Guidi S, Capurro V, Viganò D, et al. Changes in hippocampal morphology and neuroplasticity induced by adolescent THC treatment are associated with cognitive impairment in adulthood. Hippocampus. 2009;19(8):763–72. doi: 10.1002/hipo.20554 19156848

[36] Murray CH, Huang Z, Lee R, de Wit H. Adolescents are more sensitive than adults to acute behavioral and cognitive effects of THC. Neuropsychopharmacology. 2022;47(7):1331–8. doi: 10.1038/s41386-022-01281-w 35110688 PMC9117219

[37] Almog S, Aharon-Peretz J, Vulfsons S, Ogintz M, Abalia H, Lupo T, et al. The pharmacokinetics, efficacy, and safety of a novel selective-dose cannabis inhaler in patients with chronic pain: A randomized, double-blinded, placebo-controlled trial. Eur J Pain. 2020;24(8):1505–16. Epub 2020/05/24. doi: 10.1002/ejp.1605 ; PubMed Central PMCID: PMC7496774 disclosures; Dr. Hayon, Mrs. Ogintz, Mrs. Abalia, and Mr. Lupo are employees of Syqe Medical; Dr. Vulfsons and Prof. Eisenberg received research support from Syqe Medical. 32445190 PMC7496774

[38] Karniol IG, Shirakawa I, Kasinski N, Pfeferman A, Carlini EA. Cannabidiol interferes with the effects of delta 9—tetrahydrocannabinol in man. Eur J Pharmacol. 1974;28(1):172–7. Epub 1974/09/01. doi: 10.1016/0014-2999(74)90129-0 . 4609777

[39] Zuardi AW, Shirakawa I, Finkelfarb E, Karniol IG. Action of cannabidiol on the anxiety and other effects produced by delta 9-THC in normal subjects. Psychopharmacology (Berl). 1982;76(3):245–50. Epub 1982/01/01. doi: 10.1007/BF00432554 . 6285406

[40] Silva GD, Del Guerra FB, de Oliveira Lelis M, Pinto LF. Cannabidiol in the Treatment of Epilepsy: A Focused Review of Evidence and Gaps. Front Neurol. 2020;11:531939. Epub 2020/11/17. doi: 10.3389/fneur.2020.531939 ; PubMed Central PMCID: PMC7604476. 33192966 PMC7604476

[41] Varni JW, Stucky BD, Thissen D, Dewitt EM, Irwin DE, Lai JS, et al. PROMIS Pediatric Pain Interference Scale: an item response theory analysis of the pediatric pain item bank. J Pain. 2010;11(11):1109–19. Epub 2010/07/16. doi: 10.1016/j.jpain.2010.02.005 ; PubMed Central PMCID: PMC3129595. 20627819 PMC3129595

[42] Yu L, Buysse DJ, Germain A, Moul DE, Stover A, Dodds NE, et al. Development of short forms from the PROMIS™ sleep disturbance and Sleep-Related Impairment item banks. Behav Sleep Med. 2011;10(1):6–24. Epub 2012/01/19. doi: 10.1080/15402002.2012.636266 ; PubMed Central PMCID: PMC3261577. 22250775 PMC3261577

[43] Varni JW, Magnus B, Stucky BD, Liu Y, Quinn H, Thissen D, et al. Psychometric properties of the PROMIS ® pediatric scales: precision, stability, and comparison of different scoring and administration options. Qual Life Res. 2014;23(4):1233–43. Epub 2013/10/03. doi: 10.1007/s11136-013-0544-0 ; PubMed Central PMCID: PMC4312615. 24085345 PMC4312615

[44] Irwin DE, Stucky B, Langer MM, Thissen D, Dewitt EM, Lai JS, et al. An item response analysis of the pediatric PROMIS anxiety and depressive symptoms scales. Qual Life Res. 2010;19(4):595–607. Epub 2010/03/10. doi: 10.1007/s11136-010-9619-3 ; PubMed Central PMCID: PMC3158603. 20213516 PMC3158603

[45] Gordon JE, Powell C, Rockwood K. Goal attainment scaling as a measure of clinically important change in nursing-home patients. Age Ageing. 1999;28(3):275–81. Epub 1999/09/04. doi: 10.1093/ageing/28.3.275 . 10475864

[46] Varni JW, Sherman SA, Burwinkle TM, Dickinson PE, Dixon P. The PedsQL Family Impact Module: preliminary reliability and validity. Health Qual Life Outcomes. 2004;2:55-. doi: 10.1186/1477-7525-2-55 . 15450120 PMC521692

[47] Birnie KA, Hundert AS, Lalloo C, Nguyen C, Stinson JN. Recommendations for selection of self-report pain intensity measures in children and adolescents: a systematic review and quality assessment of measurement properties. Pain. 2019;160(1):5–18. Epub 2018/09/05. doi: 10.1097/j.pain.0000000000001377 . 30180088

[48] Hanish AE, Lin-Dyken DC, Han JC. PROMIS Sleep Disturbance and Sleep-Related Impairment in Adolescents: Examining Psychometrics Using Self-Report and Actigraphy. Nurs Res. 2017;66(3):246–51. Epub 2017/04/28. doi: 10.1097/NNR.0000000000000217 ; PubMed Central PMCID: PMC5426113. 28448375 PMC5426113

[49] Donnelly A, Fitzgerald A, Shevlin M, Dooley B. Investigating the psychometric properties of the revised child anxiety and depression scale (RCADS) in a non-clinical sample of Irish adolescents. J Ment Health. 2019;28(4):345–56. Epub 2018/02/16. doi: 10.1080/09638237.2018.1437604 . 29447056

[50] Forrest CB, Ravens-Sieberer U, Devine J, Becker BD, Teneralli R, Moon J, et al. Development and Evaluation of the PROMIS(®) Pediatric Positive Affect Item Bank, Child-Report and Parent-Proxy Editions. J Happiness Stud. 2018;19(3):699–718. Epub 2018/05/16. doi: 10.1007/s10902-016-9843-9 ; PubMed Central PMCID: PMC5947961. 29760578 PMC5947961

[51] Harris PA, Taylor R, Thielke R, Payne J, Gonzalez N, Conde JG. Research electronic data capture (REDCap)—a metadata-driven methodology and workflow process for providing translational research informatics support. Journal of biomedical informatics. 2009;42(2):377–81. doi: 10.1016/j.jbi.2008.08.010 18929686 PMC2700030

[52] Grant CN, Belanger RE. Cannabis and Canada’s children and youth. Paediatr Child Health. 2017;22(2):98–102. Epub 2018/02/27. doi: 10.1093/pch/pxx017 ; PubMed Central PMCID: PMC5804770. 29480902 PMC5804770

[53] Baron EP, Lucas P, Eades J, Hogue O. Patterns of medicinal cannabis use, strain analysis, and substitution effect among patients with migraine, headache, arthritis, and chronic pain in a medicinal cannabis cohort. J Headache Pain. 2018;19(1):37. Epub 2018/05/26. doi: 10.1186/s10194-018-0862-2 ; PubMed Central PMCID: PMC5968020. 29797104 PMC5968020

[54] Poudel S, Quinonez J, Choudhari J, Au ZT, Paesani S, Thiess AK, et al. Medical Cannabis, Headaches, and Migraines: A Review of the Current Literature. Cureus. 2021;13(8):e17407. Epub 2021/10/01. doi: 10.7759/cureus.17407 ; PubMed Central PMCID: PMC8459575. 34589318 PMC8459575

[55] Öst E, Nisell M, Burgos CM, Frenckner B, Öjmyr-Joelsson M. Behavioral, emotional and social functioning in children born with congenital diaphragmatic hernia. Pediatr Surg Int. 2018;34(6):653–61. Epub 2018/04/11. doi: 10.1007/s00383-018-4266-9 ; PubMed Central PMCID: PMC5954068. 29637256 PMC5954068

[56] Gibbard M, Mount D, Rassekh SR, Siden HH. Family attitudes about and experiences with medical cannabis in children with cancer or epilepsy: an exploratory qualitative study. CMAJ Open. 2021;9(2):E563–e9. Epub 2021/05/23. doi: 10.9778/cmajo.20200212 ; PubMed Central PMCID: PMC8177908. 34021014 PMC8177908

[57] Schwela D. Risk Assessment, Uncertainty. In: Wexler P, editor. Encyclopedia of Toxicology (Third Edition). Oxford: Academic Press; 2014. p. 165–71.

