# Supplementary material for: A multi-centre, tolerability study of a cannabidiol-enriched Cannabis Herbal Extract for chronic headaches in adolescents: The CAN-CHA protocol
Source: PLoS One. 2024 Sep 20;19(9):e0290185. doi: 10.1371/journal.pone.0290185 (PMC11414974; doi:10.1371/journal.pone.0290185)
Supplement: S1 Checklist — (PDF) [file pone.0290185.s001.pdf]

Spirit Checklist

		Reporting Item	Page and Line Number	Reason if not applicable
**Administrative information**				
Title	#1	Descriptive title identifying the study design, population, interventions, and, if applicable, trial acronym	Page 1,Line number 1-2	
Trial registration	#2a	Trial identifier and registry name. If not yet registered, name of intended registry	Page 17, Line number 20	
Trial registration: data set	#2b	All items from the World Health Organization Trial Registration Data Set	n/a	We have registered trial with clinicaltrial.gov only
Protocol version	#3	Date and version identifier	Page 17, Line number 22	
Funding	#4	Sources and types of financial, material, and other support	Page 2, Line 25-26	
Roles and responsibilities: contributorship	#5a	Names, affiliations, and roles of protocol contributors	Page 1-2, Line 4-38, 1-11	
Roles and responsibilities: sponsor contact information	#5b	Name and contact information for the trial sponsor	Page 5, line 40	
Roles and responsibilities: sponsor and funder	#5c	Role of study sponsor and funders, if any, in study design; collection, management, analysis, and interpretation of data; writing of the report; and the decision to submit the report for publication, including whether they will have ultimate authority over any of these activities	n/a	Sponsor and funder doesn't have any of these roles in our trial

Roles and responsibilities: committees	#5d	Composition, roles, and responsibilities of the coordinating centre, steering committee, endpoint adjudication committee, data management team, and other individuals or groups overseeing the trial, if applicable (see Item 21a for data monitoring committee)	Supplementary page1 Line 1-28	
**Introduction**			Page 5-6	
Background and rationale	#6a	Description of research question and justification for undertaking the trial, including summary of relevant studies (published and unpublished) examining benefits and harms for each intervention	Page 5, Line 1-39, Page 2, Line 1-4	
Background and rationale: choice of comparators	#6b	Explanation for choice of comparators	n/a	we are not having any comparator
Objectives	#7	Specific objectives or hypotheses	Page 6, line 6-21	
Trial design	#8	Description of trial design including type of trial (eg, parallel group, crossover, factorial, single group), allocation ratio, and framework (eg, superiority, equivalence, non-inferiority, exploratory)	Page 8, Line 8-25	
**Methods: Participants, interventions, and outcomes**				
Study setting	#9	Description of study settings (eg, community clinic, academic hospital) and list of countries where data will be collected. Reference to where	Page: 16, Line 9-10	

		list of study sites can be obtained		
Eligibility criteria	#10	Inclusion and exclusion criteria for participants. If applicable, eligibility criteria for study centres and individuals who will perform the interventions (eg, surgeons, psychotherapists)	Page 6, 7 Line 22-39, 1,19	
Interventions: description	#11a	Interventions for each group with sufficient detail to allow replication, including how and when they will be administered	Page 7, 8 Line 29-40, 1-8	
Interventions: modifications	#11b	Criteria for discontinuing or modifying allocated interventions for a given trial participant (eg, drug dose change in response to harms, participant request, or improving / worsening disease)	Page 10, Line 16-36	
Interventions: adherance	#11c	Strategies to improve adherence to intervention protocols, and any procedures for monitoring adherence (eg, drug tablet return; laboratory tests)	Page 14, Line 13-41	
Interventions: concomitant care	#11d	Relevant concomitant care and interventions that are permitted or prohibited during the trial	n/a	We are not restricting concomitant care and interventions
Outcomes	#12	Primary, secondary, and other outcomes, including the specific measurement variable (eg, systolic blood pressure), analysis metric (eg, change from baseline, final value, time to event), method of aggregation (eg, median, proportion), and time point	Page 11, Line 1-37	

		for each outcome. Explanation of the clinical relevance of chosen efficacy and harm outcomes is strongly recommended		
Participant timeline	#13	Time schedule of enrolment, interventions (including any run-ins and washouts), assessments, and visits for participants. A schematic diagram is highly recommended (see Figure)	Table 1, Page 19	
Sample size	#14	Estimated number of participants needed to achieve study objectives and how it was determined, including clinical and statistical assumptions supporting any sample size calculations	Page 16, Line 4-10	
Recruitment	#15	Strategies for achieving adequate participant enrolment to reach target sample size	Page 8, Line 14-22	
**Methods: Assignment of interventions (for controlled trials)**				
Allocation: sequence generation	#16a	Method of generating the allocation sequence (eg, computer-generated random numbers), and list of any factors for stratification. To reduce predictability of a random sequence, details of any planned restriction (eg, blocking) should be provided in a separate document that is unavailable to those who enrol participants or assign interventions	n/a	Its an open label, single arm study

Allocation concealment mechanism	#16b	Mechanism of implementing the allocation sequence (eg, central telephone; sequentially numbered, opaque, sealed envelopes), describing any steps to conceal the sequence until interventions are assigned	n/a	Its an open label, single arm study
Allocation: implementation	#16c	Who will generate the allocation sequence, who will enrol participants, and who will assign participants to interventions	n/a	Its an open label, single arm study
Blinding (masking)	#17a	Who will be blinded after assignment to interventions (eg, trial participants, care providers, outcome assessors, data analysts), and how	n/a	Its an open label, single arm study
Blinding (masking): emergency unblinding	#17b	If blinded, circumstances under which unblinding is permissible, and procedure for revealing a participant's allocated intervention during the trial	n/a	Its an open label, single arm study
**Methods: Data collection, management, and analysis**				
Data collection plan	#18a	Plans for assessment and collection of outcome, baseline, and other trial data, including any related processes to promote data quality (eg, duplicate measurements, training of assessors) and a description of study instruments (eg, questionnaires, laboratory tests) along with their reliability and validity, if known. Reference to where data collection forms can be found, if not in the protocol	Page 16, Line 12-27	

Data collection plan: retention	#18b	Plans to promote participant retention and complete follow-up, including list of any outcome data to be collected for participants who discontinue or deviate from intervention protocols	Page 16, Line 12-27	
Data management	#19	Plans for data entry, coding, security, and storage, including any related processes to promote data quality (eg, double data entry; range checks for data values). Reference to where details of data management procedures can be found, if not in the protocol	Page 16, Line 12-27	
Statistics: outcomes	#20a	Statistical methods for analysing primary and secondary outcomes. Reference to where other details of the statistical analysis plan can be found, if not in the protocol	Page 16, Line 29-41	
Statistics: additional analyses	#20b	Methods for any additional analyses (eg, subgroup and adjusted analyses)	Page 16, Line 29-41	
Statistics: analysis population and missing data	#20c	Definition of analysis population relating to protocol non-adherence (eg, as randomised analysis), and any statistical methods to handle missing data (eg, multiple imputation)	n/a	It is not relevant to our study
**Methods: Monitoring**				
Data monitoring: formal committee	#21a	Composition of data monitoring committee (DMC); summary of its role and reporting structure; statement of whether it is independent from the	Supplementary page1 Line 1-28	

		sponsor and competing interests; and reference to where further details about its charter can be found, if not in the protocol. Alternatively, an explanation of why a DMC is not needed		
Data monitoring: interim analysis	#21b	Description of any interim analyses and stopping guidelines, including who will have access to these interim results and make the final decision to terminate the trial	Supplementary page1 Line 1-28	
Harms	#22	Plans for collecting, assessing, reporting, and managing solicited and spontaneously reported adverse events and other unintended effects of trial interventions or trial conduct	Supplementary page1 Line 1-28	
Auditing	#23	Frequency and procedures for auditing trial conduct, if any, and whether the process will be independent from investigators and the sponsor	Supplementary page1 Line 1-28	
**Ethics and dissemination**				
Research ethics approval	#24	Plans for seeking research ethics committee / institutional review board (REC / IRB) approval	Page 17 Line 26-30	
Protocol amendments	#25	Plans for communicating important protocol modifications (eg, changes to eligibility criteria, outcomes, analyses) to relevant parties (eg, investigators, REC / IRBs, trial participants, trial registries, journals, regulators)	n/a	No amendments

Consent or assent	#26a	Who will obtain informed consent or assent from potential trial participants or authorised surrogates, and how (see Item 32)	Page 8, Line 28-31	
Consent or assent: ancillary studies	#26b	Additional consent provisions for collection and use of participant data and biological specimens in ancillary studies, if applicable	Page 15, Line 35-38	
Confidentiality	#27	How personal information about potential and enrolled participants will be collected, shared, and maintained in order to protect confidentiality before, during, and after the trial	Page 14, Line 23-27	
Declaration of interests	#28	Financial and other competing interests for principal investigators for the overall trial and each study site	Page 2-3 Line 30-35, 1,29	
Data access	#29	Statement of who will have access to the final trial dataset, and disclosure of contractual agreements that limit such access for investigators	Page 17 Line 32-34	
Ancillary and post trial care	#30	Provisions, if any, for ancillary and post-trial care, and for compensation to those who suffer harm from trial participation	Page 8, Line 23-24	
Dissemination policy: trial results	#31a	Plans for investigators and sponsor to communicate trial results to participants, healthcare professionals, the public, and other relevant groups (eg, via publication, reporting in results databases, or other data sharing	n/a	No restrictions on publication

		arrangements), including any publication restrictions		
Dissemination policy: authorship	#31b	Authorship eligibility guidelines and any intended use of professional writers	n/a	We are not utilizing professional writers
Dissemination policy: reproducible research	#31c	Plans, if any, for granting public access to the full protocol, participant-level dataset, and statistical code	n/a	Available on request
**Appendices**				
Informed consent materials	#32	Model consent form and other related documentation given to participants and authorised surrogates	Appendix 2	
Biological specimens	#33	Plans for collection, laboratory evaluation, and storage of biological specimens for genetic or molecular analysis in the current trial and for future use in ancillary studies, if applicable	Page 13, Line 21-31	

It is strongly recommended that this checklist be read in conjunction with the SPIRIT 2013 Explanation & Elaboration for important clarification on the items. Amendments to the protocol should be tracked and dated. The SPIRIT checklist is copyrighted by the SPIRIT Group under the Creative Commons "Attribution-NonCommercial-NoDerivs 3.0 Unported" license. This checklist can be completed online using https://www.goodreports.org/, a tool made by the EQUATOR Network in collaboration with Penelope.ai
