# Supplementary material for: A multi-centre, tolerability study of a cannabidiol-enriched Cannabis Herbal Extract for chronic headaches in adolescents: The CAN-CHA protocol
Source: PLoS One. 2024 Sep 20;19(9):e0290185. doi: 10.1371/journal.pone.0290185 (PMC11414974; doi:10.1371/journal.pone.0290185)
Supplement: S1 Data — (PDF) [file pone.0290185.s002.pdf]

A Multi-Centre, Tolerability Study of a Cannabidiol-enriched Cannabis Herbal Extract for Chronic Headaches in Adolescents: the CAN-CHA trial

Protocol Number: CAN-CHA

Principal Investigator: Dr. Lauren E Kelly

Sponsor: University of Manitoba

Funded by: SickKids Foundation; Canadian Institutes of Health Research

Version Number: 6.0

01 Mar 2024

NCT 05337033

GENERAL INFORMATION

SPONSOR:

The University of Manitoba
66 Chancellors Cir, Winnipeg, MB R3T 2N2

LEAD PRINCIPAL INVESTIGATOR:

Dr. Lauren E. Kelly
Associate Professor,
Departments of Pharmacology and Therapeutics, Rady Faculty of Health Sciences, University of Manitoba

STUDY MONITORING:

Cross-Institutional Monitoring Program, Maternal Infant Child and Youth Research Network

STUDY COORDINATING CENTRE:

Canadian Collaborative for Childhood Cannabinoid Therapeutics (C4T)

Investigator Agreement

Protocol Title: A Multi-Centre, Tolerability Study of a Cannabidiol-enriched Cannabis Herbal Extract for Chronic Headaches in Adolescents: the CAN-CHA trial

Protocol Number: CAN-CHA

Version number and version date: v6.0 dated March 1, 2024

Signature of Site Qualified Investigator (QI):
I have read this protocol and agree that it contains all the necessary details for carrying out this study. I will conduct the study as outlined herein and will complete this study within the time designated.

I will provide copies of the protocol and all pertinent information to all individuals responsible to me who assist in the conduct of this study. I will discuss this material with them to ensure that they are fully informed and trained regarding the study drug, the conduct, and the obligations of confidentiality as per the Canadian Privacy Act, The Personal Information Protection and Electronic Documents Act ("PIPEDA") and the relevant HealthCare Privacy Legislations.

I agree to the terms of this protocol and all amendments. I confirm that I will conduct this clinical trial in compliance with the Health Canada Food and Drug Regulations, Part C, Division 5, the International Council for Harmonisation Good Clinical Practice Guideline (ICH-GCP E6), the Tri-Council Policy Statement: Ethical Conduct for Research Involving Humans (TCPS-2), the Protocol as approved, all applicable local and study specific standard operating procedures (SOPs), and national, provincial and local regulations.

Site Name: ____

______________________	____________________________	____________________
**QI (printed name)**	**QI's signature**	**(date)**

Table of Contents

1. STATEMENT OF COMPLIANCE 6
1 PROTOCOL SUMMARY 7
1.1 Synopsis 7
1.2 Schema 11
1.3 Schedule of Activities (SoA) 12
2 INTRODUCTION 14
2.1 Study Rationale 14
2.2 Background 14
3 Risk/Benefit Assessment 24
3.1 Known Potential Risks 24
3.2 Known potential Benefits 24
4 OBJECTIVES AND ENDPOINTS 25
5 STUDY DESIGN 28
5.1 Overall Design 28
5.2 Justification for Dose 30
6 STUDY POPULATION 30
6.1 Inclusion Criteria 30
6.2 Exclusion Criteria 31
6.3 Lifestyle Considerations 31
6.4 Screen Failures 32
6.5 Strategies for Recruitment and Retention 32
7 STUDY INTERVENTION 32
7.1 Study Intervention(s) Administration 32
7.1.1 Study Intervention Description 32
7.1.2 Dosing and Administration 32
7.2 Preparation/Handling/Storage/Accountability 34
7.2.1 Acquisition and accountability 34
7.2.2 Formulation, Appearance, Packaging, and Labeling 34
7.2.3 Product Storage and Stability 34
7.2.4 Preparation 34
7.3 Measures to Minimize Bias: Randomization and Blinding 35
7.4 Study Intervention Compliance 35
7.5 Concomitant Therapy 35
7.5.1 Rescue Medicine 35
8 STUDY INTERVENTION DISCONTINUATION AND PARTICIPANT DISCONTINUATION/WITHDRAWAL 36
8.1 Dose limiting toxicity and Discontinuation of Study Intervention 36
8.2 Participant Discontinuation/Withdrawal from the Study 37
8.3 Lost to Follow-Up 37
9 STUDY ASSESSMENTS AND PROCEDURES 38
9.1 Measures of Effectiveness 38
9.2 Safety and Other Assessments 41
9.3 Adverse Events and Serious Adverse Events 41
9.3.1 Definition of Adverse Events (AE) 41
9.3.2 Definition of Serious Adverse Events (SAE) 41
9.3.3 Classification of an Adverse Event 42
9.3.3.1 Severity of Event 42

9.3.3.2 Relationship to Study INTERVENTION 42
9.3.3.3 Expectedness 43
9.3.4 Time Period and Frequency for Event Assessment and Follow-Up 43
9.3.5 Adverse Event Reporting 43
9.3.6 Serious Adverse Event Reporting 44
9.3.7 Reporting Events to Participants 44
9.3.8 Reporting of Pregnancy 44
10 STATISTICAL CONSIDERATIONS 44
10.1 Statistical Hypotheses 44
10.2 Sample Size Determination 44
10.3 Populations for Analyses 44
10.3.1 Safety Analyses 45
10.3.2 Baseline Descriptive Statistics 45
10.3.3 Sub-Group Analyses 45
10.3.4 Tabulation of Individual participant Data 45
11 SUPPORTING DOCUMENTATION AND OPERATIONAL CONSIDERATIONS 46
11.1 Regulatory, Ethical, and Study Oversight Considerations 46
11.1.1 Informed Consent Process 46
11.1.1.1 Consent/assent and Other Informational Documents Provided to participants 46
11.1.1.2 Consent and Assent Procedures and Documentation .. 46
11.1.1.3 Study Discontinuation and Closure 46
11.1.2 Confidentiality and Privacy 47
11.1.3 Future Use of Stored Specimens and Data 47
11.1.4 Key Roles and Study Governance 48
11.1.5 Safety Oversight 48
11.1.6 Clinical Monitoring 49
11.1.7 Quality Assurance and Quality Control 49
11.1.8 Data Handling and Record Keeping 50
11.1.8.1 Data Collection and Management Responsibilities 50
11.1.9 Protocol Deviations 50
11.1.10 Conflict of Interest Policy 51
11.2 Abbreviations 51
11.3 Protocol Amendment History 52
12 REFERENCES 53

1. STATEMENT OF COMPLIANCE

This document is a protocol for a clinical research trial study involving the human participants. The trial will be conducted in accordance with International Conference on Harmonisation Good Clinical Practice (ICH GCP E6 (R2)), Health Canada Division 5 (Part C, Division 5 of the Food and Drug Regulations “Drugs for Clinical Trials Involving Human Subjects”), and applicable regulatory requirements.

The protocol, informed consent form(s), recruitment materials, and all participant materials will be submitted to the REB for review and approval. Approval of both the protocol and the consent form must be obtained before any participant is enrolled. Any amendment to the protocol will require review and approval by the REB before the changes are implemented to the study. All changes to the consent form will be REB approved; a determination will be made regarding whether a new consent needs to be obtained from participants who provided consent, using a previously approved consent form.

1 PROTOCOL SUMMARY

1.1 SYNOPSIS

Title	A Multi-Centre, Tolerability Study of a Cannabidiol-enriched Cannabis Herbal Extract for Chronic Headaches in Adolescents: the CAN-CHA trial
Short Title	Cannabis for Chronic Headaches in Adolescents (CAN-CHA)
Protocol Number	CAN-CHA
Version Number	6.0
Phase	2
Study Drug	MediPharm Labs MPL-001, CBD:THC 50:2 (50mg of CBD per ml to 2mg of THC per ml, dissolved in medium chain triglycerides oil derived from fractionated palm oil) with a lemon peppermint flavouring 1500mg of total CBD per 30ml bottle
Treatment Regimen	MediPharm Labs MPL-001, CBD:THC 50:2, will be used as a study Intervention. Study participants will receive escalating doses of CBD starting at 0.2-0.4 mg/kg/day up to 0.8-1 mg/kg/day followed by a weaning period. Dosed twice a day orally
Study Design	A multicenter, open-label, dose-escalation study will be conducted across three centres in Canada to study the safety, tolerability, and effectiveness of a CBD-enriched CHE in adolescents with chronic headaches. The study will consist of four different phases: recruitment, baseline (1 month), treatment (4 months) and weaning (1 month).
Study Duration	The participants will be enrolled in the study for 6 months and complete 7 study visits. The overall study is expected to be completed within 24 months.<br>Recruitment/Screening<br>Baseline: 1 month, Treatment: 4 months, Weaning: 1 month
Objectives	**Primary Objective**: To determine the safety and tolerability of escalating doses of a Cannabidiol (CBD)-enriched Cannabis Herbal Extract (CHE) in adolescents with chronic headaches.<br><br>**Secondary Objectives:**<br>1. To investigate the relationship between the dose-escalation with headache-free days.

	2. To monitor the effect of CBD-enriched CHE oil on the intensity of pain related to chronic headaches.<br>3. To evaluate the effect of CBD-enriched CHE oil on sleep, mood and function in adolescents with chronic headaches.<br>4. To explore the impact of chronic headaches on quality of life<br>**Exploratory Objectives:**<br>1. To investigate the relationship between the dose-escalation and steady-state trough levels of bioactive cannabinoids.<br>2. To investigate the relationship between pharmacogenetic variations and response outcomes in adolescents receiving CBD-enriched CHE oil.
Endpoints	**Primary Endpoint:**<br>The frequency and type of treatment-emergent adverse events among participants treated with CBD-enriched CHE reported daily and evaluated monthly before the subsequent dose escalation<br>**Secondary Endpoints:**<br>1. Number of headache-free days per month.<br>2. Percentage change in average daily pain intensity due to headache on the numeric rating scale (NRS) from baseline to each follow up visit.<br>3. Sleep, mood and function are to be reported using the percentage change in the scores of PROMIS (patient-reported outcome measurement information system) measures from the Pediatric Item Bank:<br>a. Pediatric Sleep-related Impairment – Short Form 8a<br>b. Pediatric Short Form v2.0 - Anxiety - 8a<br>c. Pediatric Short Form v2.0 - Depressive Symptoms 8a<br>d. Pediatric Pain Interference – Short Form 8a<br>e. Pediatric Positive Affect – Short Form 8a<br>f. Self-directed goal attainment will be reported as a percentage toward a physical, mental and social goal reported by participants at each monthly visit.<br>**4.** Family impact scores will be reported by PedsQL™ Family Impact Module, Version 2.0 and compared with baseline scores.

	**Exploratory Endpoints:**<br>5. Steady-state plasma levels of bioactive cannabinoids, namely THC, CBD, 11-OH-THC, 7-OH-CBD, and Cannabichromene, during each follow up visit before dose escalation.<br>6. Genetic polymorphisms of genes encoding for CYP enzymes and the p-glycoprotein transporter and plasma levels of THC, CBD and their active metabolites in the study participants.
Number of Participants	20 participants
Inclusion Criteria	Adolescent patients of the qualified investigator between the ages of 14-17 years with chronic headaches will be enrolled.<br>They must have headaches (migraine or tension-like features) on more than 15 days per month for more than 3 months and have failed at least two treatment options for either tolerability or efficacy.<br>Participants must be committed to using contraception, abstaining from recreational cannabis use, and working with psychology and physiotherapy teams as appropriate.
Statistical Methodology	*Sample Size determination:*<br>Precedent suggests that a power calculation is not appropriate for a dose-finding study. The study site and the number of planned dose escalations are standard for clinical trials.<br>*Populations for Analyses:*<br>We will complete an intent to treat analysis. All participants who received the investigational product for at least one month will be included in the analysis to ensure that these data would be likely to represent the effects of the study intervention.<br>*Safety Analyses:*<br>The severity, frequency, and relationship of AEs to study intervention will be presented by System Organ Class (SOC). The start date, stop date, severity, relationship, expectedness, outcome, and duration of each AE will be reported. Serious treatment-emergent AEs will be presented either in a table or a listing.<br>*Baseline and Descriptive Statistics:*<br>We will perform the descriptive statistics to describe baseline characteristics and other outcomes. The proportions and frequencies will be used to categories the categorical variable.

	The mean, median, range or standard deviations will be used to categories the continuous endpoints. Subgroup summarization based on dose level or other criteria may also be conducted. Median concentrations of CBD, THC and the major metabolites will be reported with ranges for each sampling time point. The ratio of parent to metabolites will be reported to provide estimates of the variability in cannabinoid metabolism.<br>*Sub-Group Analyses:*<br>We will perform prespecified subgroup analysis of primary and secondary outcomes-based sex and dose.

1.2 SCHEMA

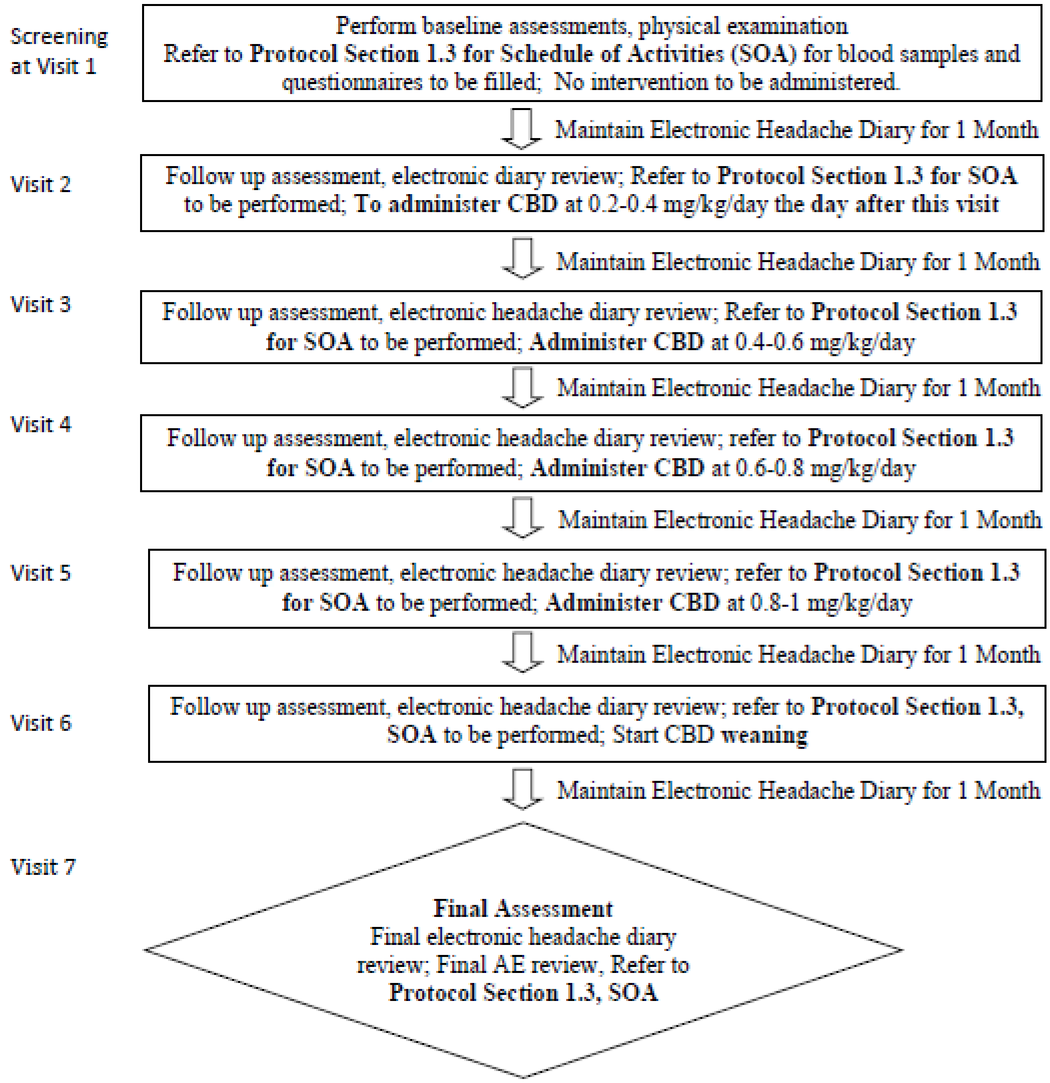

Figure 1: A flow chart of participant enrollment, treatment with CBD-Enriched CHE, monitoring and weaning

1.3 SCHEDULE OF ACTIVITIES (SOA)

Table 1. CAN-CHA schedule of activities

Study visit #	**1 (Baseline)**	**2**	**3**	**4**	**5**	**6**	**7**
Day #	**1**	**30**	**60**	**90**	**120**	**150**	**180**
Follow up window (days)	**±5**	**± 5**	**± 5**	**±5**	**± 5**	**±5**	**± 5**
Informed Consent	X						
Demographics	X						
Medical history (safety screening with SCR Checklist)[b]	X						
Review ongoing co-existing medical conditions[d]		X	X	X	X	X	X
Concomitant medication and therapy review	X	X	X	X	X	X	X
IP intervention follow-up[a]		X[a]	X[a]	X[a]	X[a]	X[a]	
Intervention dose of CBD (mg/kg/day)		0.2 - 0.4	0.4 - 0.6	0.6 - 0.8	0.8-1.0	Wean	
Physical exam	X						X
Vital signs and weight[c]	X	X	X	X	X	X	X
Pregnancy test in females[e]	X	X	X	X	X	X	X
Assign Daily eDiary via REDCap	X						
Review of Daily Diary		X	X	X	X	X	X
Review of Adverse Events		X	X	X	X	X	X
Review of Cannabis-related Adverse Event		X	X	X	X	X	X
Cannabis Use Disorder Identification Test Short-Form (CUDIT-SF)	X						X
Pediatric Sleep-Related Impairment– Short Form 8a Scale	X	X	X	X	X	X	X
PedsQL Family Impact	X	X	X	X	X	X	X
Pediatric Short Form v2.0 - Anxiety - 8a scale	X	X	X	X	X	X	X
Pediatric Short Form v2.0 - Depressive Symptoms 8a scale	X	X	X	X	X	X	X

Pediatric Pain Interference – Short Form 8a scale	X	X	X	X	X	X	X
Pediatric Positive Affect – Short Form 8a	X	X	X	X	X	X	X
Self-directed goal attainment	X	X	X	X	X	X	X
Participant Trial Expectation Survey	X						X
Blood samples (8mLs, approx.)	X	X	X	X	X	X	X
Saliva Sample for Pharmacogenomics	X						
Electrocardiogram (if not done in the last 3 months)	X						
Drug Accountability		X	X	X	X	X	X
Visit duration (minutes)	60	45	60	45	60	45	60

a. If participant withdraw from the study intervention prior to visit 7 but consents to stay in the study, they will be followed up till visit 7 for all the other assessments, including bloodwork, eDiary and questionnaires. Site study team will provide guidance on IP administration and follow up with participant regarding their experience of IP administration at home. IP administration will not be completed at study site during the scheduled study visit.

b. Document titled "C4T Baseline Safety Screening Tool for Cannabis-related interventional trials", also called "SCR Checklist" will be used by study team to document thorough medical history at visit 1, with Common Terminology Criteria for Adverse Events (CTCAE) grade level to be applied with each reported medical history condition.

c. Participants will self-administer the investigational product orally following a dosing calendar and all dosing calculations will be based on weight at baseline (visit 1), unless there is a significant body weight change that would affect dosing. If participant's body weight increases or decreases for 10% or above comparing with baseline data, site investigator will contact study PI Dr. Lauren Kelly for further instructions regarding dose change or participant withdrawing. The decision will be made on a case-by-case basis to protect participant's safety, and in the meanwhile minimize negative impact on data integrity.

d. Any ongoing co-existing medical condition at visit 1 will be followed up by site study team at follow-up visits to document changes.

e. Both urine and blood pregnancy tests will be completed at visit 1. Blood pregnancy test will be completed from visit 2- visit 7.

2 INTRODUCTION

2.1 STUDY RATIONALE

Randomized Control Trials (RCTs) and open-label studies have demonstrated the well-defined margin of safety and efficacy of CBD and CBD-enriched CHE in children with intractable epilepsy.[1-9] Chronic headaches are one of the most common pain syndromes in adolescence, and despite numerous treatment options, roughly only half of adolescents with Chronic Headache (C) experience remission. CBD and other cannabis products are being used off-label for the treatment of chronic headaches. [10] Prescription cannabinoids (nabiximols) and other cannabis-based products containing THC are currently being used off-label in adolescents with chronic headaches. The overall purpose of this open-label phase 2 study is to generate clinical evidence on the safety, and tolerability of a CBD-enriched CHE in adolescents suffering from chronic headaches. Furthermore, the ideal tolerated dose derived from this study will inform a future RCT to explore the efficacy of CBD-enriched CHE in a larger cohort. In this study, participants will receive escalating doses to a maximum of 1.0mg/kg of CBD/day to treat the chronic headaches. There have been many historical challenges with studying cannabis products and cannabinoids due to difficulties in consistently obtaining products, variable quality control and stigma associated with cannabis. Canada has the opportunity to be a world leader in cannabis research. The successful outcomes from this research project have the potential to create a meaningful impact on the lives of Canadian adolescents, given the high prevalence of chronic headaches, severe morbidity, and the current lack of effective treatment options.

2.2 BACKGROUND

Globally, chronic headaches are significant cause of disability among adolescents; the World Health Organization classifies chronic headaches under the top ten disabling health conditions.[11] [12] In children and adolescents the prevalence of chronic headaches is 7.8%, higher than in the general population (3-4%).[13-15] The chronic headache is a common debilitating disorder, where headaches occur for 15 days per month, for at least 3 months.[16] chronic headaches give rise to distress, disruption of daily life activities, and significant out-of-pocket expenditures to families and societies by increasing healthcare costs. [16] The term chronic headaches refer to the primary headache disorders, namely Chronic Migraine (CM), Chronic Tension-Type Headaches (CTTH), Medication Overuse Headache (MOH) and New Daily Persistent Headache (NDPH) after the head trauma or infection.[11]

Adolescents with the chronic headaches suffer from reduced quality of life, sleep disruption, anxiety, absenteeism from school, fatigue, limb pain, dizziness, overuse of medications and school failure.[17] The International Classification of Headache Disorders (ICHD) further classifies migraine into two subtypes; migraine with aura and migraine without aura.[17] ICHD defines migraine with aura as a recurring headache disorder which lasts between 4-72 hours characterized by the pulsating headache of unilateral location, severe to moderate intensity accompanied by nausea with or without photophobia and phonophobia.[17] Migraine without aura is defined as the headache

with recurrent attacks characterized by the continuous minutes of unilateral auditory, tactile or other manifestations of the central nervous system that generally developed slowly and are accompanied by headache and related migraine symptoms.[17] Chronic TTH is defined as lasting for >15 days for at least six months characterized by somatic symptoms such as difficulty in getting and staying asleep, irritability, generalized muscle pain and joint pain, impaired memory and focus.[18] MOH is defined as a headache lasting for 15 or more days per month as a result of regular overuse of acute or symptomatic medications for ten or more, for more than three months.[19] NDPH is defined as the new onset of headache in the person without a history of persistent headache; it persists for more than three months. Patients with NDPH do remember the time or the conditions when the headache originated. [20]

Despite the advancements in the therapeutic management of chronic pain, the treatment of chronic headaches in adolescents remains to be challenging. Non-steroidal anti-inflammatory drugs (NSAIDs) are used for the treatment of mild headaches.[21] While, antidepressants, triptans, ergotamine and verapamil are used for the severe type of headaches with very higher pain intensity.[22] Further, amitriptyline, topiramate and gabapentin are used as maintenance therapy, but their use is off label in children. Only the small open-label studies with limited safety and efficacy evidence advocate their use in children with headaches. [23]

Treatment decisions for the most part in adolescent patients with chronic headaches are made based on data from the studies conducted in adult patients. [24] Nevertheless, Cannabis demonstrates promising results in adolescents where traditional medications and therapies have not worked, especially for treatment-resistant epilepsy and headache. [25] An observational study reported that more than a quarter of the cannabis users rely on it for relieving their symptoms related to migraines/headaches. Further, they reported an average decrease of 3.6 points in the intensity of headache on a scale of 10 after the use of cannabis. [26] There is paucity in the literature demonstrating its efficacy and safety for the treatment of chronic headaches. The current scenario warrants the need to conduct interventional studies establishing the safety and efficacy of cannabis in adolescents with chronic headaches.

Epidemiology of Chronic Headache

The prevalence of chronic headache among the general population is around 4%,[27] however, the epidemiology of chronic headache is highly influenced by the age group, sex, socioeconomic factors, ethnicity, geographical regions and its subtypes .[28] Females are more likely to suffer from chronic headache as compared to males.[29, 30] Abu-Arafeh et al conducted a systematic review of 50 population-based observational studies conducted on children and adolescents.[31] They reported the prevalence of headache and/or migraine in the covered population between the one-month and lifetime in adolescents and children as high as 58.4% [CI: 58.1-58.8]. [31] The prevalence of chronic headache is 2-3 times higher in females than in males, even during the preadolescence period. [32] The prevalence of probable migraine affects 17% of female and 5.6% of males in older adolescents >15 years.[33] As per the estimates of multiple prevalence studies, 31% (10-72%) of children with chronic headaches suffer from CTTH.[12] Furthermore, the prevalence of MOH ranges between 1-1.5%.[34] In the general population, the prevalence of the NDPH ranges from 0.03 to

0.1%.[12] However, its prevalence is higher among children and adolescents as compared to adults.[12]

Chronic headaches and comorbid conditions

The literature supports that adolescents suffering from chronic headaches are more likely to suffer from sleep disruptions, mood disorders like depression, school absenteeism, anxiety, and pain in the abdomen.[35, 36] In addition, primary headaches, especially migraines, are associated with other comorbid conditions such as attention deficit hyperactivity disorder, anxiety, depression, stroke and epilepsy.[37] Adolescents suffering from chronic headaches suffers from sleep disruptions characterized by inadequate total sleep, excessive sleepiness during the daytime, difficulty falling and staying asleep.[37, 38] Approximately 25% of the children suffering from migraines experience at least one type of sleep problem.[39] Generally, the association between headache and sleep conditions may be influenced by pain. A broad survey of 622 children and adolescents with pain (60% with headache) recorded that the most prevalent pain-related symptoms were sleep disruptions (53.6%), accompanied by a failure to practice daily activities (53.3%), food difficulties (51.1%), and absenteeism from school.[40] A cross-sectional study conducted in the USA showed that 65.7% of the adolescents with the primary headache had experienced reduced sleep duration of <8 hours during the school nights.[41]] Furthermore, they also reported that only 10.4% of the adolescents had a sleeping duration of ≤ 6 hours. However, only 4.3% of the adolescents were able to sleep for the ideal sleeping hours. They also found a statistically significant association between the higher intensity of pain due to headache with the prolonged time for the onset of sleep. Additionally, they also reported the significant association of nightmares in adolescents with frequently occurring headaches.[41]

Management of Chronic Headaches

The management of chronic headaches is challenging, and it may take a few weeks to months to get a positive therapeutic outcome in controlling headaches. The successful management of chronic headaches consists of “three-prong approach", physical therapy, psychotherapy and pharmacotherapy.[11, 24, 42] The treatment of adolescents with chronic headaches starts with a clear-cut description and diagnosis, reassurance for lack of severe etiology and predisposing factors. Before initiating the therapy, the recommended monitoring of lifestyle issues is dietary/sleep habits, exercise schedule, weight, and caffeine intake. Addressing the chronic headache’s triggering factors, such as loud music and bright sunlight, relieves the patient. Both pharmacological and non-pharmacological options are available for the management of headaches in adolescents.[24] The most commonly used non-pharmacological therapy for the management of chronic headaches in adolescents is Cognitive Behavioral Therapy (CBT).[42] The medications used to treat headaches are classified of two types; abortive medications and prophylactic medications.[43] Abortive medications are used for the acute management of headache, irrespective of the intensity of the pain and frequency. While prophylactic medications are used to prevent/ reduce the episodes of headache attacks.[43] The NSAIDs and triptans are used as abortive medication; on the other hand, antiepileptic drugs, antihypertensives (beta-blockers) and antidepressants are used as prophylactic therapy. However, pharmacological agents

like NSAIDS are proven to be ineffective for the treatment of chronic headaches. [43] There are very few randomized studies to guide physicians on safe and effective options for the treatment of chronic headaches in adolescents, as such almost all existing therapies for children are off label. Studies in the children have shown that tricyclic antidepressants are adequate for the management of headaches in some cases.[44, 45] However, there are some challenges as they require periodical therapeutic drug monitoring and electrocardiogram (ECG) for safety, and tricyclic antidepressants have been associated with weight gain in adolescents. Selective Serotonin Reuptake Inhibitors, calcium channel blockers, nonsteroidal anti-inflammatory drugs and beta-blockers are also used as prophylactic medication in the childhood headache. [44-46] There are certain demerits of these agents, which cannot be overlooked in adolescents. The use of beta-blockers is associated with nightmares and sadness,[47] calcium channel blockers result in orthostatic hypertension and constipation.[48] Acetaminophen and Aspirin are the less costly interventions available for treating the chronic headaches in children. Still, they are nephrotoxic in chronic high doses, and Aspirin is more likely to cause Reye's syndrome in children and teenagers ≤18 years of age. [49,50, 51] Analgesics/NSAIDs cannot be used for prophylactic use, and they are not recommended for chronic pain. Chronic use of analgesic agents results in rebound headaches and medication overuse. [44] There is a need to explore the promising therapeutic agents for the treatment of chronic headaches. Furthermore, commonly used intravenous medications for the chronic headaches are dihydroergotamine, valproate and dexamethasone, [44] these medications have been shown to relieve the pain, but they can be administered in hospital settings only. This warrants the strong need to explore the safety and efficacy of available potential outpatient therapeutic options for the management of chronic headaches in adolescents.

Cannabis for the treatment of Chronic Headache

Cannabis is a complex plant with many different active components. Clinical literature on the effectiveness of cannabis-based products for headache is summarized in Table 2. Cannabinoids can be administered in a variety of routes including inhalation, oral and sublingual routes or can be made with tea and mixed with edible items.[25, 52]. Cannabidiol (CBD) and Delta-9 Tetrahydrocannabinol (THC) are the two primary (and most abundant) active cannabinoids. [53] The direct mechanism by which cannabinoids work to modulate headache response is poorly understood. CBD acts as a negative allosteric modulator on the cannabinoid CB1 receptors and decreases the potencies of exogenous agonists (including THC) and 2-arachidonoyl-sn-glycerol (2-AG, an endocannabinoid).[54] CBD also potentiates anandamide mediated intrinsic neurotransmission.[55]

Table 2. Clinical literature on Cannabis for the treatment of headache

Author	Year	Location	Study design	Population	Findings
El-Mallakh et al [56]	1987	USA	Case series	Patient with a history of chronic smoking and migraine	Patients complain of headache after discontinuation of marijuana cigarettes; one patient got relief from headache after reinitiating cannabis smoking
Russo E et al. [10]	2001	USA	Case series	Patient with a history of migraine	After the use of medical Cannabis, the patient experienced superior relief from headache as compared to ergots, opiates and beta-blockers
Grinspoon and Bakalar [57]	1997	USA	Case report	Patient with a history of treatment failure for migraine	The patient got relieved from migraine with smoked Cannabis
Rhyne DN et al. [58]	2016	0USA	Retrospective, Observational chart review	Patients with migraine headaches on medical Cannabis therapy	Patients had a decrease in the headache episodes with the migraine therapy (10.4% to 4.6% monthly), reduction in frequency (19.8% decrease) and abortion of pain in 11.6% of the study participants
Mikuriya et al [59]	1991	USA	Case series	Patients with a history of	All the cases were treated with either

				chronic migraine treated with cannabis/ Dronabinol	Cannabis or Dronabinol. Cannabis was found to be more efficacious as compared to the Dronabinol
Nunberg et al. [60]	2011	USA	Medical record analysis and patient survey	Patients presenting to the Medical Marijuana clinic	The significant improvement in headache symptoms was reported by the participants who were on Cannabis.
Pini et al. [61]	2012	Italy	A randomized controlled trial, Nabilone	Patients with Medication Overuse headache for last five years	Nabilone was found to be superior as compared to Ibuprofen in terms of intake of analgesics, reducing pain intensity.

Preclinical studies on Cannabis for pain

Pain presents as a common and debilitating symptom for adolescents with chronic headache. Cannabinoids have demonstrated promising efficacy in various models of neuropathic, chronic inflammatory pain, and multiple acute pain models of rodents such as tail-flick, hot plate and formalin test. [62-68] The preclinical studies shed the evidence of antinociceptive effects of the CBD and/or THC agonists acting typically on CB1 receptors, CB2 receptors or both CB1 and CB2 receptors; theses agonists are believed to reduce the neuroinflammation part of neuropathic pain. [66, 69-79] Nevertheless, the evidence claims the presence of expression of the CB2 receptor on the neuronal cells. [67, 80, 81] The stimulation of CB2 receptors in the midbrain leads to dopamine release from the nerve cell of the ventral tegmental area. [82-84] Furthermore, this leads to analgesia and inhibits descending pain control.[85, 86] Primarily, the peripherally acting cannabinoids cause analgesic effects without any specific cannabinoid-induced behavioural side effects in the rodent species. [70, 87]

Additionally, their efficiency was also attested with the compounds that prevent the reuptake of the cannabinoids present in the body (endocannabinoids), [88] thus by elevating the endocannabinoid content synaptically or preventing the degradation of 2-arachidonoylglycerol and Anandamide. [89-92] Generally, preclinical shreds of evidence advocate the antinociceptive effects of the monoacylglycerol lipase (degrader pf 2-AG), fatty acid amide hydrolase (FAAH) and anandamide degrading enzymes, these evidence help to understand the reverse pain sustaining the action of these components. [89, 93-97] Compounds related endocannabinoids such as

palmitoylethanolamide and oleoylethanolamide, that are not direct agonists on the CB1 and CB2 receptors, have shown analgesic potential in the rodent models of neuropathic pain, inflammatory pain and visceral pain. [65, 88, 98, 99] Possibly, they have shown their therapeutic effects via peroxisome proliferator-activated receptor gamma (PPARs), blocking the transient receptor potential cation channel subfamily V 1 (TRPV1), negatively regulating the Fatty Acid Amide Hydrolase (FAAH) and also by acting on the nonneuronal cells.[100-102] . The antinociceptive effect of CBD has been studied extensively in rodents to manage the visceral pain associated with multiple sclerosis, parkinsonism, intractable cancer pain. [103-105] CBD acts as an antagonist at CB1/2 and on the brain membranes of the rat at 8.6 ±0.2 micromole/L of minimum effective concentration. The mechanisms may include nuclear receptor stimulation, atypical cannabinoid receptor antagonism such as the GPR55, calcium flux regulation through the TRP channels.[106] Moreover, a preclinical study report that there is either downregulation or internalization of CB1 receptors resulting in resistance of cannabinoid agonist. However, resistance and withdrawal symptoms were significantly less in the rodents who were on opioids. [107]

Previous experience with cannabinoids

CBD is frequently used off-label to treat a wide variety of conditions including Chronic Pain, Multiple Sclerosis, Parkinson disease, Schizophrenia, Generalized Anxiety Disorder and Crohn’s disease.[108, 109] In clinical trials with adults, the total daily dose of CBD ranges between 20-1000 mg of CBD per day. [5, 110, 111] Table 3 describes the evidence-based literature of interventional studies of cannabis products in children.

Additionally, Epilepsy is the most studied condition for evaluating the effect of CBD in children. Four RCTs have been conducted in pediatric patients investigating the efficacy of CBD. [5, 112-114] and Epidiolex ® (99% pure cannabidiol) is approved by Food and Drugs Administration (FDA) and regulators globally as an add on therapy for the treatment of Lennox–Gastaut Syndrome (LGS) and Dravet Syndrome (DS) in the patient aged > 2 years. [115] The dose range of CBD used in the RCTs was ranging between 5- 20 mg/kg/d. [5, 112-114] Moreover, it was well tolerated in the studied subjects for more than 24 months of follow-up, while only in a few patients, elevations in the liver transaminase enzymes and somnolence was reported.[5, 113, 114] Cannabidiol in Children with Refractory Epileptic Encephalopathy (CARE-E) in an ongoing study exploring the efficacy and tolerability of escalating doses of CBD enriched herbal extract in the patient suffering from treatment-resistant epileptic encephalopathy.[116] The CARE-E trial investigates the tolerance, safety and efficacy of CBD-enriched CHE (1:25 THC:CBD) in reducing the frequency and duration of seizures and quality of life.[116] The interim results of the CARE-E included seven patients reported that a 1:25 CHE was well tolerated at a dose of 10-12mg kg/day.[117]

Table 3: Clinical literature demonstrating the efficacy of Cannabis in the pediatric population

Author and year	Disease/ Condition	Study design and location	Age group and sample size	Duration of treatment	Intervention	Comparator	Findings
Devinsky O et al. 2018 [113]	Dravet syndrome	Phase 3; Multisite, randomized, double-blind, placebo-controlled, parallel-group trial; the United Kingdom and the United States	4-10 years; 34	3-week treatment period; ≤ 10 days of tapering	CBD: 5 mg/kg/d; CBD: 10; CBD: 20 mg/kg/d	Placebo	There was a dose-proportional increase in the concentration of CBD and its metabolite. As a result, more adverse events were observed with CBD, but all were well tolerated.
Devinsky O et al. 2018 [5]	Lennox–Gastaut syndrome	Phase 3, Parallel-group, randomized, placebo-controlled trial USA, Spain, UK, France	2 to 55 years; 225	14-week treatment ≤ ten days of tapering	CBD: 5 mg/kg/d; CBD: 10; CBD: 20 mg/kg/d	Placebo	CBD was found to be efficacious in controlling the seizure, and it was associated with elevated transamin

							ases levels
Devinsky O et al. 2017 [114]	Dravet syndrome	Multinational, randomized, double-blind trial; the United States and Europe	2 to 18 years; 120	4-week treatment period; 14 days titration, 12-week maintenance therapy, ≤ Ten days of tapering	CBD: 5 mg/kg/d; CBD: 10; CBD: 20 mg/kg/d	Placebo	There was a significant reduction in the seizures among the patients who were on CBD as compared to the placebo
Thiele E A et al. [112]	Lennox-Gastaut syndrome	Randomized, double-blind, placebo-controlled trial; USA, Netherlands, Poland	2–55 years; 171	14 weeks -week, two weeks of titration phase, 12-weeks of maintenance therapy, ≤ Ten days of tapering	20 mg/kg/d	Placebo	CBD at a dose of 20 mg/kg/day was found to be safe and well-tolerated by the patients

Study Product

The product used in our study will be MPL-001 (containing 50mg of CBD and 2mg of THC per ml), a full spectrum cannabis concentrate (oil). The product is packaged in a pharmaceutical-grade amber glass bottle with child-resistant cap, with a 1mL syringe for consistent dosing. MediPharm Labs uses strict manufacturing and quality standards. MPL-001 must be stored at room temperature (15 to 25 °C). It should be protected from direct sunlight and avoid freezing.

Risk of Cannabis Dependence Screening

During the design of this trial, the study team conducted focus groups with youth around medical cannabis adverse event reporting. Youth were asked to share their questions about medical cannabis products and shared concerns about the risk of dependence. Given their priority for this question, and the lack of data available to inform public health on the risk of cannabis dependence in youth when introducing them to high-CBD

cannabis product, the Cannabis Use Disorder Identification Test Short-Form (CUDIT-SF) will be administered to trial participants at visit 1 and end-of-study visit.

CUDIT-SF is developed from the Cannabis Use Disorder Identification Test – Revised (CUDIT-R). [139] CUDIT-R was validated among college students and young adults (under 26 yrs). [136,137,138] Results indicate that CUDIT-R would validly distinguish between young adults with and without CUD and may be useful in screening for CUD in this high-risk population. [136,137] CUDIT-SF is a short form contains 3 questions from CUDIT-R (8 questions); study has demonstrated that the CUDIT-SF can quickly screen CUD in busy clinical setting while reducing the questionnaire administration burden on both clinicians and clinic patients. [139]

<u>CUDIT-SF used in this trial contains the following questions:</u>

Outside of the oil product that you are taking in this study, do you currently use cannabis? YES/NO

IF YES:

1. How often during the past 6 months did you find that you were not able to stop using cannabis once you had started?

Never	Less than monthly	Monthly	Weekly	Daily or almost daily
0	1	2	3	4

2. How often in the past 6 months have you devoted a great deal of your time to getting, using, or recovering from cannabis?

Never	Less than monthly	Monthly	Weekly	Daily or almost daily
0	1	2	3	4

3. How often in the past 6 months have you had a problem with your memory or concentration after using cannabis?

Never	Less than monthly	Monthly	Weekly	Daily or almost daily
0	1	2	3	4

The CUDIT-SF will not be used as an exclusion screening tool. If participant agrees not to use recreational cannabis during their trial participation, they should not be excluded from the trial.

Participant who scores 2 or higher will be considered a positive screen. Site study staff will notify the treating physician (site Qualified Investigator) when there is a positive screen (score above 2) and clinical care plans for managing potential cannabis use disorder will be followed.

3 RISK/BENEFIT ASSESSMENT

3.1 KNOWN POTENTIAL RISKS

The use of Cannabinol (CBD) and Tetrahydrocannabinol (THC) is associated with some risk of developing adverse effects of the Central Nervous System (CNS) and peripheral organ system. The most common side effects related to CBD and THC extracts are described below. Literature suggests that the incidence of less common side effects with Cannabis extract is lower in long-term studies compared to short-term (e.g., single dose) studies, which is because of the development of tolerance.

The Adverse Drug Reactions (ADRs) associated with the use of CBD and THC are described below.

>10%

Drowsiness (23-25%); general infections (21-25%); decreased appetite (16-22%); fatigue, weakness, and lack of energy (11-12%); diarrhea (9-20%); liver damage (8-16%); rash (7-13%); insomnia (5-11%)

1-10%

Irritability, agitation (5-9%); Pneumonia (5-8%); Sedation (3-6%); Anger/ aggression (3-5%); decreased weight (3-5%); infectious diarrhea (4%); Hypoxia/Respiratory failure (3%); fungal infection (1-3%)

3.2 KNOWN POTENTIAL BENEFITS

The CBD-enriched CHE may help improve chronic headache symptoms, reduce the frequency of headaches, and improve the quality of life based on anecdotal and clinical experience. CBD-enriched CHE is hypothesized to improve sleep quality and physical functioning in patients suffering from the chronic headaches, however, there is a paucity of literature documenting the potential benefits of cannabis in the treatment of adolescents with chronic headaches.

4 OBJECTIVES AND ENDPOINTS

OBJECTIVES	ENDPOINTS	JUSTIFICATION FOR ENDPOINTS
**Primary**		
To determine the safety and tolerability of 1:25 CBD-enriched Cannabis Herbal Extract (CHE) in adolescents with chronic headaches	The frequency and type of treatment-emergent adverse events among patients treated with CBD-enriched CHE reported daily and evaluated monthly before the subsequent dose escalation<br>[Time Frame: daily throughout the study]	This is a first in human trial technically, underpowered for efficacy so primary objective is safety of CBD
**Secondary**		
To investigate relationships between the dose-escalation and change in the frequency of headache.	The percentage change in the number of headache-free days per month from baseline to each follow up visit<br>[Time Frame: monthly throughout the study]	Headache is a common symptom in patients with chronic headaches; It will be an essential measure to study the effect of CBD-enriched CHE on the incident of headache-free days
To monitor the effect of CBD-enriched CHE on the intensity of pain related to chronic headache	The percentage reduction in the average daily pain intensity due to chronic headache on the Numeric Rating Scale (NRS) from baseline to each follow up visit.<br>[Time Frame: daily throughout the study]	It will be helpful to evaluate the effect of CBD –enriched CHE on the intensity of pain related to chronic headache
To evaluate the effect of CBD-enriched CHE on sleep, mood and function in adolescents with chronic headaches.	Sleep, mood, and function are to be reported using the percentage change in the scores of PROMIS	The pain and discomfort in chronic headaches are associated with the decrease in the

	(Patient Reported Outcome Measurement Information System) measures from the pediatric Item bank:<br><br>a. Pediatric Sleep-Related Impairment– Short Form 8a scale<br>b. Pediatric Short Form v2.0 – Anxiety – 8a scale<br>c. Pediatric Short Form v2.0 – Depressive Symptoms 8a scale<br>d. Pediatric Pain Interference – Short Form 8a scale<br>e. Pediatric Positive Affect – Short Form 8a and goal attainment scaling (participant and parent-reported)<br>f. Self-directed goal attainment will be reported as percentage toward a physical, mental and social goal reported by participant<br><br>[Time Frame: monthly throughout the study]	quality and quantity of sleep; patients are more likely to suffer from depression and anxiety and have a functional disability, absence of school; these measures will be reasonable to determine the effect of CHE on sleep, mood and physical function
To explore the impact of chronic headaches on quality of life.	Scores of PedsQL ™ Family Impact Module Version 2.0 and compared with baseline scores<br><br>[Time Frame: monthly throughout the study]	These endpoints will be used to estimate the impact of a CBD-enriched CHE on quality of life. As cannabis products may affect a wide variety of symptoms, including pain, sleep, mood and function, quality of life provides

		an overall impact on family impact.
**Exploratory Objectives**		
To investigate the relationship between the dose-escalation and steady-state trough levels of bioactive cannabinoids	Steady-state trough levels of bioactive cannabinoids, namely THC, CBD, 11-OH-THC, 7-OH-CBD, and Cannabichromene during each follow up visit before dose escalation<br>[Time Frame: monthly throughout the study]	It is an important measure to study the linearity of the pharmacokinetics of the drug product to better understand who responds best and how metabolism changes overtime and between individuals.
To investigate the relationship between pharmacogenetic variation and response outcomes in adolescents receiving CBD-enriched CHE	Genetic polymorphisms of genes encoding for CYP enzymes and the p-glycoprotein transporter and plasma levels of THC, CBD and their active metabolites in the study participants	Cannabinoid response is highly variable. This will help to get a more clear picture of variability in seen in cannabinoid pharmacokinetic and pharmacodynamic response.

5 STUDY DESIGN

5.1 OVERALL DESIGN

This is multicenter phase 2, single group assignment, open-label dose-escalation, pharmacokinetic trial. A traditional 3+3 dose-finding design was not practical as all participants would not receive the lowest cannabinoid dose and the recommended “start low and go slow” approach to titration. The trial will be conducted across three centres in Canada and was designed in collaboration with youth from the KidsCAN Young Persons’ Advisory Group (YPAG) and the Solutions for Kids in Pain (SKIP) network. The Canadian Childhood Cannabinoid Clinical Trials Parent Advisory Committee provided insight on the outcome measurement tools and the consent form. Three youth advisors with chronic migraines have been involved throughout the study design process and will advise on recruitment strategies, reporting and dissemination as steering committee members. The youth engagement plan, findings and evaluation are described elsewhere (*publication under review)*. The research team held three virtual meetings with youth advisors to discuss which outcomes are important to youth, and decided on data collection methods and tools. The research team also co-designed the recruitment materials, including consent forms, with youth advisors. The PI will send safety reports annually to Health Canada as a Clinical Trial Notification to strengthen the evidence base of product specific cannabis data. The study will consist of three different phases: baseline (1 month), followed by escalating treatments monthly. Participants will wean in the same dosing increments over 4 weeks and will complete a pre and post study questionnaire.

Baseline Run-In Phase (from visit 1 to visit 2, the first month)

Eligible adolescents will be asked by their healthcare providers if they are interested in learning more about this research study. A study team member, who is not involved in patients’ care, will present the study, consent documents and answer any questions from the patients and their families. Participants who meet the inclusion criteria and sign the informed consent (ICF) will be enrolled in the study.

Upon enrollment, baseline visit (visit 1) will be scheduled to collect participant’s demographic data, medical history and concomitant medicine history (in the past 2 years). In addition, an electronic headache diary will be set up for participant via REDCap.

C4T Baseline Safety Screening Tool for Cannabis-related interventional trials, also called SCR Checklist, will be used at visit 1 to capture thorough medical history, along with respective CTCAE grade level to document medical condition severity level at baseline. Medical conditions reported as medical history at baseline should not be reported as AE during the study, unless increases in frequency or severity. For instance: only fatigue increased in severity compared with baseline level should be reported as AE from baseline onwards.

Moreover, the baseline values (collected at visit 1) of each experimental measure will be estimated prior to administering the intervention/study product to the participants on visit 2. The participant will be asked to maintain the electronic headache diary, reporting headache-free days encountered by the participants, and will be asked to fill the

following age validated scales (described above) on sleep related impairment, mood, and life impact. Participants will select three goals and work through goal attainment scaling each visit.

A handout on cannabis oil administration will be co-created with the youth advisors and will be provided to all study participants and their families. There will also be a demonstration by the research coordinator at the first study visit. On visit 1, the blood sample will be drawn from the study participants to measure the plasma concentration of CBD, THC and its metabolites and saliva sample for extraction of genomic DNA to allow for genotyping of pharmacogenetic variants. ECG (within 3 months of screening or at screening) will be recorded for all the participants to ensure participants do not have any concerning changes in cardiac electrical activity. All participants will undergo the lab investigation for liver transaminases (ALT/AST) and creatinine levels for all follow-up visits. All the study participants will be provided with an option of using wrist-worn device (optional) for actigraphy to monitor their sleep parameters. Participant will be advised that if they choose to accept and wearing the actigraphy device, data generated on the device won't be collected or analyzed for study purpose.

Treatment Phase (visit 2 to visit 5)

Before visit 2 (start of the second month) the study participants will receive CBD-enriched CHE (MPL-001) and a dosing calendar shipped to their homes before visit 2. Participants will be instructed to administer it at a dose of 0.2-0.4 mg/kg of CBD per day divided into two doses (25% in the morning and 75% in the afternoon after school). Participant-specific dose will be prescribed by site investigator. Participants will be asked to administer CHE with food.

Participants will complete monthly study visits and receive a one monthly supply of MPL-001. They will be instructed on how many drops to administer daily for the following one-month period. Participants will be asked to administer the intervention for the next month at escalating doses of 0.4-0.6 mg/kg/day then 0.6-0.8 mg/kg/day at the visit 4, and finally 0.8-1.0 mg/kg/day at visit 5. Blood samples will be drawn each month to measure the plasma concentration of CBD, THC, and its metabolites before increasing the dose, pregnancy test and liver and renal safety lab test. For all the follow-up visits, participants will be asked to provide information about PedsQL ™ Family Impact Module assessment and perception of improvement, self-directed goal attainment scaling.

Weaning Phase (from visit 6 to visit 7)

After the baseline phase (one month) and the treatment phase (four months), participants will start the weaning schedule, which comprises incrementally reducing the dose leading to complete discontinuation of the study product. The intervention dose will be decreased in 0.2mg/kg increments every week. The intervention will be discontinued by visit 7. If the parents, adolescents, and healthcare providers feel an improvement in chronic headache, participants will be offered a medical cannabis authorization at the end of the study.

All participants and their caregivers will be invited to complete a post-study interview (telephone) about their experiences in the trial to inform future research. Questions will be asked around their overall impression of the trial, perceived risks, benefits, how it changed during the trial, outcome importance for future studies, challenges they encountered, and how we can improve our trial. More specific questions will also be asked on the usability and usefulness of the consent form, recruiting materials, outcomes measured and the daily headache collection tool.

5.2 JUSTIFICATION FOR DOSE

Data on the pharmacokinetics related to the active constituent of Cannabis, i.e., CBD and THC, in adolescents are lacking. The CBD dose is extrapolated from safety data obtained in clinical trials in children with refractory epilepsy in the current study.[1-7, 118] Moreover, we made our present considerations about the dosing regimen based on the nature of our study product, namely an extract containing one of the major biologically active cannabinoids but also with pharmacologically relevant levels of other cannabinoids such as THC and cannabichromene.[119] CBD was found to be safe and well-tolerated in children with refractory epilepsy at a dose of 10-20mg kg/day. The suggested dose of CBD in the phase 2 study of refractory epilepsy was 10 mg/kg/day. [1, 4, 5, 7, 113, 118] The use of THC is associated with some risk of developing adverse effects on the central nervous system and peripheral organ system. However, these appear to be mitigated by the coadministration of CBD. THC possesses its significant pain-relieving potential at very low doses. [120] The quantity of THC in our study product is 2mg per mL, with maximum THC doses only reaching 0.05mg/kg/day, which we do not expect to be associated with any adverse psychoactive reactions. In this study, the maximum dose of CBD will be less than 10 percent of the recommended dose of the CBD in the previous studies conducted in the pediatric population. We aim to keep the CBD dose as low as possible to reduce costs for families to confirm a tolerable dose and demonstrate efficacy in future clinical trials.

6 STUDY POPULATION

6.1 INCLUSION CRITERIA

To be eligible to participate in this study, an individual must meet all of the following criteria:

1. Adolescents aged between 14-17 years of age at the time of screening.
2. Diagnosed with Chronic Migraine according to ICHD-3: headache (migraine-like or tension-type like) occurring on 15 or more days per month for more than 3 months which on at least 8 days per month have features of migraine headache. [121]
3. Failed at least two treatment options on the grounds of safety (tolerability) and/or efficacy, including but not limited to antidepressant (tricyclic antidepressant or selective norepinephrine reuptake inhibitor), magnesium, gabapentinoids topiramate and/or non-pharmacological therapies.
4. Females who have reached menarche should have a negative pregnancy test during screening.

5. Must be willing to engage with psychology and physiotherapy throughout the trial as appropriate.

6.2 EXCLUSION CRITERIA

An individual who meets any of the following criteria will be excluded:

1. As per the investigator judgement, the participant is not an ideal candidate due to a personal issue or medical condition that is likely to impede in the successful completion of the study
2. Participants with a history of post-concussion headache or new daily persistent headache
3. Participants with a diagnosis of medication overuse headache
4. Participants with cardiac, renal or hepatic disease (assessed by the site investigator)
5. Participants with complex regional pain syndrome-II
6. Participants with abnormal ECG findings at baseline (as determined by the investigator)
7. Participants who are on the following medications: opioids, antipsychotics, antimanic, barbiturates, benzodiazepines, muscle relaxants, sedatives, or tramadol.
8. Participants with developmental delay or impairments including autism, cerebral palsy or intellectual disability.
9. Participants with a personal or family history of schizophrenia or psychotic disorders
10. Participants who are pregnant or breast/chest-feeding or plans to become pregnant within the study period or within three months of interventional product discontinuation
11. Participants who cannot commit to using contraception and refraining from recreational cannabis use and driving throughout the study period
12. Participants with known allergy to cannabinoids and/or palm/coconut oil

6.3 LIFESTYLE CONSIDERATIONS

Participants must agree to not drive during the study and the study visits. This is an absolute risk avoidance approach for the safety of participants and the public in the face of very little evidence supporting the safety of cannabidiol use while operating a motor vehicle. Participants will be asked to use contraception and will include monthly pregnancy tests. Female participants on hormonal contraceptives should be advised to use an additional alternative, non-hormonal/reliable barrier method of birth control during the study. Male, female and nonbinary participants will all be encouraged to continue contraceptive precautions for up to three months after discontinuation of

cannabis-based therapies. Recreational cannabis use during the study will be discouraged.

6.4 SCREEN FAILURES

Screen failures are defined as participants who consent to participate in the clinical trial but are not subsequently assigned to the study intervention or entered into the study. A minimal set of screen failure information is required to ensure the transparent reporting of screen failure participants. Minimum information includes demographic details, screen failure details, eligibility criteria, and any serious adverse events (SAEs).

6.5 STRATEGIES FOR RECRUITMENT AND RETENTION

We plan to recruit 20 participants satisfying our inclusion criteria across the three chronic pain programs; Halifax, Vancouver, and Toronto. All three programs at each site manage around > 10 adolescent patients with chronic headaches in a month. Our study's sample size and dose escalation are coherent with the standard of phase 2 clinical trials. Potential participants presenting to the pain clinics across all three centres will be approached to participate in the study.

If the adolescent or parent is interested in participating in the study, a research coordinator will contact them. To improve retention, we co-developed our consent form with youth advisors. To compensate participants for their time and encourage daily data entry participants will be compensated up to $105 for the study participation, which is $15 per completed study visit, and 0.50 per daily diary entry. Adolescents and the C4T youth advisors were asked how to improve recruitment and retention into CAN-CHA. They recommended consistent follow-up and personal check-ins from the study team if a daily entry was missed for more than 2 days in a row which will be implemented in this trial.

7 STUDY INTERVENTION

7.1 STUDY INTERVENTION(S) ADMINISTRATION

7.1.1 STUDY INTERVENTION DESCRIPTION

This study's investigational product is oil-based CBD enriched CHE, MediPharm Labs MPL-001 (CBD:THC 25:1) oil. MPL-001 is available in 30ml bottles where each ml of oil contains 2 mg of THC and 50 mg of CBD dissolved in coconut/palm-based medium chain triglyceride (MCT) carrier oil. The high-quality formulated oil has a unique flavour profile from the naturally occurring cannabis terpenes. The MPL-001 (CBD:THC 25:1) oil used in this study contains lemon-peppermint flavouring agents. Manufacturing of MPL-001 (CBD:THC 25:1) oil occurs following Good Manufacturing Practices.

7.1.2 DOSING AND ADMINISTRATION

Eligible study participants will complete a baseline phase (first month) where no investigational product will be administered for one month. At the four subsequent study visits, study participants will receive escalating doses of CBD-enriched CHE from 0.2-0.4 mg/kg/day to 0.8-1 mg/kg/day for four months, with dose increases happening

monthly over four months in 0.2mg increments. Participants will self-administer the investigational product orally following a dosing calendar and all dosing calculations will be based on weight at baseline. The product comes as CHE oil, 25% of the daily dose will be administered in the morning, and the remaining 75% of the dose in the afternoon to mimic endogenous cannabinoid diurnal variation. Upon completing the baseline phase, the eligible study participants will receive a supply of study products delivered to their home from the study central pharmacy at Children's Hospital Research Institute of Manitoba (CHRIM). Each package will include a participant-specific dosing calendar for dose confirmation, and a return envelope to the central study pharmacy at CHRIM for used bottles to be mailed back for destruction and accountability.

During visit 2, study participants will be instructed how to administer the investigational product (IP) at a dose of 0.2-0.4 mg/kg/day divided into two doses (BID, 25% dose in morning and 75% in the afternoon) for one month. Participant's day 1 of IP dose starts the day after visit 2.

After one month of the intervention, the participants will receive new supply/supplies of the investigational product from CHRIM during visits 3 through 5 (See table 4). The 6th month will include a weekly weaning period where the dose is reduced each week at the same rate as the dose escalation (0.2mg/kg/day) (See table 5). To maintain the accuracy and consistency in the dosing regimen of the study participants across all the study centres, the mid-point of the dose range will be selected to calculate the desired dose based upon the weight of the participants. Participants will be weighed on site at each study visit. However, the dose calculation will be based on the weight taken at baseline. The final dose will be calculated by rounding off the nearest 1 mg of CBD. As an exemplary 24 kg, (0.3mg/kg/day) adolescent will receive a dose of 7 mg/day. This will help achieve improved efficacy, precision in dosing and ease the administration of the MPL-001 (CBD:THC 25:1) oil to the study participants.

Table: 4 The single escalating dose of the MediPharm Labs MPL-001 Oil from visit 2 to visit 5

Visit	Dose	Frequency	Duration
Visit 2	0.2-0.4 mg/kg/day	BID	1 month
Visit 3	0.4-0.6 mg/kg/day	BID	1 month
Visit 4	0.6-0.8 mg/kg/day	BID	1 month
Visit 5	0.8-1 mg/kg/day	BID	1 month

Weaning of MediPharm Labs MPL-001 Oil

At visit 6, participants and their parents will be instructed on how to wean the investigational product starting from 0.8-0.6 mg/kg/day, incrementally decreasing the dose of administered study product over time (See Table 5) in weekly increments. If the adolescents and clinicians feel that there was an improvement in chronic headache, participants will be offered medical cannabis authorization to access a 1:25 THC:CBD product at the end of the study.

Table: 5 The weaning schedule of the MediPharm Labs MPL-001 Oil at visit 6

Visit 6	Dose	Frequency	Duration
Week 1	0.6-0.8 mg/kg/day	BID	7 days
Week 2	0.4-0.6 mg/kg/day	BID	7 days
Week 3	0.2-0.4 mg/kg/day	BID	7 days
Week 4	0 mg/kg/day	BID	7 days
*Visit 7: Assessment of ADR, electronic headache diary, blood samples, Score of questionnaires, out of pocket expenditures, quality of life score			

7.2 PREPARATION/HANDLING/STORAGE/ACCOUNTABILITY

7.2.1 ACQUISITION AND ACCOUNTABILITY

Our study ensures strict compliance with Health Canada's requirements for conducting the clinical research study involving the cannabis product. Special care is taken to assure accountability concerning the investigational product dispensed and utilized by the study participant. Investigational product will be shipped to participants in batches and accountability documented on the dosing calendar will be reviewed with the study team at each monthly visit. The MediPharm Labs MPL-001 Oil will be shipped, stored, dispensed, destroyed and documented according to the trial specific site SOPs.

7.2.2 FORMULATION, APPEARANCE, PACKAGING, AND LABELING

MediPharm Labs MPL-001 Oil is packaged in a pharmaceutical-grade amber glass bottle with a 1mL syringe for consistent dosing. Study dosage and protocol number will be marked on the bottles.

7.2.3 PRODUCT STORAGE AND STABILITY

The study product is packaged in 30 mL amber bottles with child-resistant caps. The bottles must be stored at room temperature (15 to 25 °C).

The study participants will be instructed to cap the bottle after use and keep it away from direct sunlight.

7.2.4 PREPARATION

MediPharm Labs MPL-001 Oil will be made ready to be dispensed as oil bottles. The study team at each site will be given rigorous training to prevent any dispensing errors.

7.3 MEASURES TO MINIMIZE BIAS: RANDOMIZATION AND BLINDING

This is a non-randomized, open-label tolerability study.

7.4 STUDY INTERVENTION COMPLIANCE

The study participants will be instructed to maintain the daily electronic diaries, which will help monitor the participant's compliance. During each follow-up visit, the study team will review the participant's dosing calendar. The study participants will be provided with return packaging and labels to return the unused extract or empty bottles to the study central pharmacy where it will be destroyed as per local SOPs. However, we will also draw the blood sample from the participants to measure the plasma concentration of CBD, THC, and their metabolites during all the follow-up visits starting from visit 2.

7.5 CONCOMITANT THERAPY

For this protocol, prescription medication is defined as a medication prescribed only by a duly authorized/licensed clinician. Medications and other concomitant therapies reported in the Case Report Form (CRF) will include all concomitant prescription medications, over-the-counter medications, and supplements including nutraceuticals such as magnesium. Therapies including physical and psychological will also be recorded in the daily diary.

The concomitant medications will be recorded from (Baseline phase) visit 1 until the last study visit. The participant/caregiver will be asked to record the frequency dosage and duration of concomitant medications, all use of rescue medications and will be asked to present the list during each follow-up visit.

Prohibited medications:

All opioids including tramadol, antipsychotics, antimanic meds, barbiturates, benzodiazepines, muscle relaxants, or sedatives should be avoided by participants during the entire study duration.

7.5.1 RESCUE MEDICINE

The participants will be asked to keep track of all medications during the study period. It will help to understand the influence of the study intervention, including any medicines provided as rescue medication should a visit to the emergency department be required.

8 STUDY INTERVENTION DISCONTINUATION AND PARTICIPANT DISCONTINUATION/WITHDRAWAL

8.1 DOSE LIMITING TOXICITY AND DISCONTINUATION OF STUDY INTERVENTION

Adverse events are categorized using the Common Terminology Criteria for Adverse Events (CTCAE version 5.0 dated 27 Nov 2017). If any of the following occur the participant will not move up to the next dose level (dose limiting toxicities):

1. Parental/youth report complaints of moderate mood elevation defined as exaggerated feelings of well-being which is disproportionate to the events and stimuli (Euphoria Grade 2)
2. Somnolence Grade 2 which includes moderate sedation (sleepiness and drowsiness) that limits instrumental activities of daily living
3. Cannabis-attributed diarrhea, Grade 2 or more defined as an increase of 4 - 6 stools per day over baseline; moderate increase in ostomy output compared to baseline; limiting instrumental activities of daily living
4. Unexplained tachycardia (w/out pain, fever, anemia etc.) requiring medical intervention
5. Unexplained hypotension requiring medical intervention
6. Non-infectious conjunctivitis Grade 2 defined as moderate decrease in visual acuity (best corrected visual acuity 20/40 and better or 3 lines or less decreased vision from known baseline) characterized by inflammation, swelling and redness to the conjunctiva of the eye.
7. Serious adverse events requiring hospitalization
8. Discretion of the participant, physician, or parents

Discontinuation from the study intervention does not mean interruption from the study, and remaining study procedures should be completed as indicated by the study protocol.

There will be no replacement of the study participants who were administered the investigational product, withdraw from the study, and lose follow-up (after signing an informed consent form). The main reason for the withdrawal of participants from the study will be noted in the participant's Case Record Form (CRF). The site investigator should contact the participants who fail to report during the follow-up visits which will be documented in the CRF.

Adverse events (AEs) and SAEs (Serious Adverse Events) will be reported according to Health Canada and Institutional regulations and reviewed by the data safety monitoring board (DSMB). The DSMB will hasten safety review if death is deemed related or potentially relevant to CBD-enriched CHE used in the study as the investigational product.

8.2 PARTICIPANT DISCONTINUATION/WITHDRAWAL FROM THE STUDY

Participants are free to withdraw from the study at any time. QI may discontinue or withdraw a participant from the study for the following reasons:

- The study participants suffer treatment-related intolerable adverse effects
- If the participant's headache gets worse
- The participant becomes pregnant
- The participant is unable to attend the study site for the follow-up visits
- The study participant does not comply with the prescribed regimen of the investigational drug
- The study terminated at a particular site either by site qualified investigator, principal investigator or due to any other reasons such as administrative
- If the participant meets an exclusion criterion (either newly developed or not previously recognized) that precludes further study participation

The reason for participant discontinuation or withdrawal from the study will be recorded on the CRF. Participants will be given the option to only withdraw from the study intervention, these participants will continue to be followed up till the end of study (visit 7) with the other assessments listed in SOA.

Participants who sign the informed consent form and are enrolled but do not receive the study intervention may be replaced. Study participants who sign the informed consent form and are enrolled receiving the study intervention and subsequently withdraw/discontinue will not be replaced.

8.3 LOST TO FOLLOW-UP

Participants will be considered lost to follow-up if they fail to return for all the follow-up visits and cannot be contacted by the study staff.

The following actions must be taken if a participant fails to return to the clinic for a required study visit:

- The site will attempt to contact the participant and reschedule the missed visit and counsel the participant on the importance of maintaining the assigned visit schedule and ascertain if the participant wishes to and/or should continue in the study
- Before a participant is deemed lost to follow-up, the investigator, research coordinator or designee will make every effort to regain contact with the participant (where possible, three telephone calls, and, if necessary, a certified letter to the participant's last known mailing address or local equivalent methods). These contact attempts should be documented in the participant's medical record or study file
- Should the participant continue to be unreachable, they will be considered withdrawn from the study with a primary reason for loss to follow-up

9 STUDY ASSESSMENTS AND PROCEDURES

9.1 MEASURES OF EFFECTIVENESS

Participants will participate in the study for 6 months, including the follow-up visits. Participant data will be collected and entered into electronic CRF in a Research Electronic Data Capture (REDCap) database. The following assessment will take place for the study participants:

Physical examination

During the baseline visit, the eligible study participants will undergo physical assessments, including height, weight, and calculation of Body Mass Index (BMI), evaluation of organ system, motor, and vision. Blood work will assess liver and renal safety lab tests, plasma concentration of cannabinoids and saliva samples for pharmacogenomic analyses. All the female participants who have experienced menarche will have pregnancy test at baseline and at all follow-up visits. At the time of screening, participants will receive an electrocardiogram should they not have had one in the previous 3 months. Vital signs and weight will be collected at every visit. The weight obtained at the baseline visit will be the weight used to determine the dose of investigational product throughout the study.

Electronic headache diary

The study participant will be instructed to maintain the electronic headache diary, monitor and report the information related to the intensity of headache on the NRS, and use of the rescue medication. Information about headache triggers will also be collected. The daily diary was developed in collaboration with the youth advisors. The administration will incorporate best practices in daily diaries in adolescents and feedback from patients with chronic pain and families interviewed about the use of in-home longitudinal ecological momentary assessment tools. [122] The NRS is a validated tool used to record daily self-reports of headache intensity from visit 1 until the end of the study.[123] Participants will rate the intensity of pain from 0 to 10, 0 means no hurt, 10 represents the worst hurt you could ever imagine. The efficacy of the investigational product in treating the chronic headache will be assessed in reducing the intensity of headache from baseline to each follow-up visit. REDCap will be used to complete electronic diaries. Daily data from the electronic headache diaries will be transferred to the server. Based on the intensity of the NRS, the study participant will be categorized into the severity of the headaches. The mild headache will vary in intensity between 1-5, moderate between 6-8, and severe between 9-10.[124]

Actigraphy

All the study participants will be given an option of using a wrist worn device to help them keep track of their sleep parameters. Participants will be notified that data collected on the device won’t be used in this study.

PROMIS measures

Patient-Reported Outcomes Measurement Information System (PROMIS) is the patient-centric measure that evaluates adults and children's social, mental, and physical health. [125] We will be using the PROMIS tools to measure the outcomes in the patients,

pain, sleep, depressive symptoms, feelings, and quality of life. The study participants will administer the following PROMIS measure.

Pediatric Sleep-Related Impairment- Short Form 8a scale

The pediatric sleep-related impairment- short form 8a scale is the PROMIS tool used to assess perceptions of sleepiness during usual awake hours and reported impairments during the day associated with sleep problems or daytime sleepiness. Mainly this tool estimates the sleepiness during the daytime, sleep offset, and its impact on cognitive, other activities, as well as emotional impact. The Pediatric Sleep-Related Impairment- Short Form 8a scale is a reliable generic scale with good validity and internal consistency.[126]

Pediatric Anxiety Symptoms- Short Form 8a scale v2.0

The pediatric short form v2.0 Anxiety scale is a (PROMIS) tool that assesses the self-reported fear (fearfulness, panic), anxious misery (worry, dread), hyperarousal (tension, nervousness, restlessness), and somatic symptoms related to arousal (racing heart, dizziness). The symptoms of autonomic arousal and experience of threat reflect the anxiety. The pediatric short form v2.0 Anxiety scale is non-disease specific, and it can assess the anxiety in the children over the past seven days. The literature reports the excellent reliability and validity of the Pediatric Short Form v2.0 - Anxiety - 8a scale.[127]

Pediatric Depressive Symptoms Short Form 8a scale v2.0

Pediatric Short Form v2.0 - Depressive Symptoms 8a is a PROMIS tool that is used for assessment of negative mood like sadness or guilt, self-criticism, worthlessness (self-views), loneliness, interpersonal alienation (social cognition) and decreased positive affect and engagement, which includes loss of meaning, interest and purpose. However, this scale does not assess somatic symptoms such as changes in appetite and sleeping patterns. This scale is again generic rather than disease-specific. Pediatric Short Form v2.0 - Depressive Symptoms 8a scale possesses good test-retest reliability and validity of administration across groups.[128]

Pediatric Pain Interference

The Pediatric Pain Interference, PROMIS tool assesses the self-reported consequences of pain on the essential aspects of a patient’s life, which comprises the extent by which pain interferes with activities of a person such as cognitive, emotional, social, physical, and recreational activities. The pediatric pain interference scale is generic, not focusing on any of the disease-specific conditions. The Pain Interference items utilize a 7–day recall period (items include the phrase "the past 7 days"). Varni JW et al. reported that PROMIS Pediatric Pain Interference Scale contains the item set that provides maximal test information at the mean of 50 on the T-score metric. [129]

Goal Attainment Scaling

Goal attainment scaling is a patient-reported outcome measure. Goals are selected, and further goals are standardized to extend by which the study participant meets the chosen goals. Here, we aim to choose the attainment of physical, mental and social goals. In this scaling, goals are weighted with the importance multiplied by difficulty. Further, importance and difficulty are rated on a scale of 4, starting from 0 to 3; where 0

means not at all (important/difficult). 4 means very important/difficult, leading to the generation of scoring goals. Where -1 means the patient experiences difficulties in achieving that particular goal, while -2 will be the baseline rate. [130]

Pediatric Positive Affect – Short Form 8a scale

The Pediatric Positive Affect item bank is a PROMIS tool used to assess momentary positive or rewarding affective experiences of a child. These experiences consist of feelings and moods, including elation, pride, affection, pleasure, joy, engagement, excitement, happiness, and contentment. The short form of the Pediatric Positive Affect scale is an efficient, accurate, and valid assessment of positive affect in adolescents, and good reliability has been reported. [131]

PedsQL™ Family Impact Module

The pediatric quality of life family impact module aims to measure children's chronic health conditions on families and parents. The assessments undertaken by the PedsQL are physical, emotional, social, cognitive, worry and communication. The PedsQL scale has demonstrated well-defined validity and reliability. It is the generic tool used to assess the impact of chronic disease in children on their families functioning and health-related quality of life of families. [132]

Cannabinoid and metabolite evaluation

Monthly blood samples will be collected, and routine safety liver and renal lab test will be analysed locally at satellite sites. Dose-exposure-response will compare measurable cannabinoids and active metabolites (e.g., CBD, THC, Δ9-tetrahydrocannibivarin, cannabidivarin, cannabachromene) with changes in adverse events and other secondary endpoints. We will record steady-state minimum plasma concentrations (CSS,min) before each dose escalation and at the end of treatment. Samples will be collected via venipuncture by a registered phlebotomist or research nurse, before participants take the next dose of study intervention. Samples will be sent to Dr. Jane Alcorn's lab on dry ice and stored at -70°C, for stability and to avoid cannabinoid substrate binding. Samples for cannabinoid levels will be analyzed in the Alcorn lab by a validated LC-MS/MS protocol.[116]

Pharmacogenomic analyses

Genomic DNA will be extracted from saliva samples using a QIAmp DNA extraction kit in Dr. Britt Drögemöller's lab. Genotyping will be performed using the Illumina global screening array (GSA) v3 array on the iScan array scanner. The GSAv3 GWAS arrays have been designed to capture both common and rare genetic variation, including all variants that the Clinical Pharmacogenetics Implementation Consortium has shown to affect the function of the selected candidate genes.[133] Genotyped variants and samples will undergo standard quality control procedures in PLINK 1.9. [134] Imputation of non-genotyped variants will be performed using the Michigan Imputation Server, including the Haplotype Reference Consortium data as a reference, and genetically determined ancestry will be assessed with EIGENSOFT v5. [135] Association between these variants and response outcomes will be performed using logistic regression.

Use of study data for future research

Future research may help further the understanding of the studying and other related conditions, drug response and toxicity, and can help identify new drug targets or biomarkers that predict participant response to treatment. Therefore, de-identified data collected from this study will be stored and used for future research when consented by participants unless prohibited by local laws or REBs (in such case, consent for future use of collected de-identified study data will not be included in the local ICF).

For participants who consent to the use of their de-identified study data, data may be used after the study ends for future research related either to the drug, the mechanism of action, and the studying condition or its associated conditions.

9.2 SAFETY AND OTHER ASSESSMENTS

The study's primary objective is to determine the safety and tolerability of the MediPharm Labs MPL-001 Oil, the investigational product in adolescents with chronic headache. To achieve this objective, treatment of emergent adverse events, vital signs, laboratory results, physical examinations, cannabis use disorder screening and concomitant therapy will be assessed. Study participants will be directed to record adverse events throughout the study. Self-reported information of adverse events from the patients will be reviewed with the study coordinator at each study visit.

9.3 ADVERSE EVENTS AND SERIOUS ADVERSE EVENTS

9.3.1 DEFINITION OF ADVERSE EVENTS (AE)

Adverse event means any untoward medical occurrence in a clinical study where a participant administered a medicinal product, which does not necessarily have a causal association with the treatment. An AE can therefore be any unfavourable and/or unintended sign, symptom, or disease temporally associated with the use of a medicinal product, whether or not considered related to the medicinal product. AEs may also include pre-or post-treatment complications resulting from protocol-specified procedures, overdose, drug abuse/misuse reports, or occupational exposure. In addition, pre-existing events that increase in severity or frequency or change in nature during or as a consequence of participation in the clinical study will also be considered AEs.

9.3.2 DEFINITION OF SERIOUS ADVERSE EVENTS (SAE)

An adverse event (AE) or suspected adverse reaction is considered "serious" if, in the view of either the investigator or sponsor, it results in any of the following outcomes: death, a life-threatening adverse event, inpatient hospitalization or prolongation of existing hospitalization, a persistent or significant disability/incapacity or substantial disruption of the ability to conduct normal life functions, or a congenital anomaly/birth defect.

Important medical events that may not result in death, be life-threatening or require hospitalization may be considered serious when, based upon appropriate medical judgment, they may jeopardize the participant and may require medical or surgical intervention to prevent one of the outcomes listed in this definition. Examples of such

medical events include allergic bronchospasm requiring intensive treatment in an emergency room or at home, blood dyscrasias or convulsions that do not result in inpatient hospitalization, or drug dependency or drug abuse development.

9.3.3 CLASSIFICATION OF AN ADVERSE EVENT

9.3.3.1 SEVERITY OF EVENT

The following guidelines will describe severity for adverse events (AEs) not included in the protocol defined grading system.

- **Mild** – Events require minimal or no treatment and do not interfere with the participant’s daily activities.
- **Moderate** – Events result in a low level of inconvenience or concern with the therapeutic measures. Moderate events may cause some interference with functioning.
- **Severe** – Events interrupt a participant's usual daily activity and require systemic drug therapy or other treatment. Severe events are usually potentially life-threatening or incapacitating. Of note, the term "severe" does not necessarily equate to "serious".

9.3.3.2 RELATIONSHIP TO STUDY INTERVENTION

All adverse events (AEs) must have their relationship to study intervention assessed by the clinician who examines and evaluates the participant based on temporal relationship and his/her clinical judgment. The degree of certainty about causality will be graded using the categories below. In a clinical trial, the study product must always be suspect.

- **Related** – There is clear evidence to suggest a causal relationship, and other possible contributing factors can be ruled out. The clinical event, including an abnormal laboratory test result, occurs in a plausible time relationship to study intervention administration and cannot be explained by concurrent disease or other drugs or chemicals. The response to the withdrawal of the study intervention (dechallenge) should be clinically plausible. The event must be pharmacologically or phenomenologically definitive, with use of a satisfactory rechallenge procedure if necessary.
- **Potentially Related** – There is some evidence to suggest a causal relationship (e.g., the event occurred within a reasonable time after administration of the trial medication). However, other factors may have contributed to the event (e.g., the participant’s clinical condition, other concomitant events). Although an AE may rate only as “possibly related” soon after discovery, it can be flagged as requiring more information and later be upgraded to “probably related” or “definitely related”, as appropriate.
- **Not Related** – The AE is completely independent of study intervention administration, and/or evidence exists that the event is definitely related to another etiology. There must be an alternative, definitive etiology documented by the clinician.

- **Unknown** – According to all the information available, there is not enough information for site investigator to make decision.

9.3.3.3 EXPECTEDNESS

The site investigator at each study site will be responsible for determining whether an adverse event (AE) is expected or unexpected. An AE will be considered unexpected if the nature, severity, or frequency of the event is not consistent with the risk information previously described for the study intervention.

9.3.4 TIME PERIOD AND FREQUENCY FOR EVENT ASSESSMENT AND FOLLOW-UP

The occurrence of an adverse event AE or SAE may come to the attention of study personnel during study visits and interviews of a study participant presenting for medical care or upon review by a study monitor.

All AEs, including local and systemic reactions not meeting SAEs' criteria, will be captured on the appropriate case report form (CRF) of REDCap. Information to be collected includes event description, time of onset, clinician's assessment of severity, relationship to study product (assessed only by those with the training and authority to make a diagnosis), and time of resolution/stabilization of the event. All AEs occurring while on the study must be documented appropriately regardless of relationship. All AEs will be followed to an adequate resolution.

Any medical condition present at the time that the participant is screened will be considered a baseline and not reported as an AE. However, if the study participant's condition deteriorates during the study, it will be recorded as an AE. Changes in the severity of an AE will be documented to allow an assessment of the duration of event at each level of severity to be performed. AEs characterized as intermittent require documentation of onset and duration of each episode.

The study team will record all reportable events with start dates occurring any time after informed consent is obtained and for the duration of study participation. At each study visit, the investigator will inquire about the occurrence of AE/SAEs since the last visit. Events will be followed for outcome information until resolution or stabilization.

9.3.5 ADVERSE EVENT REPORTING

All severe, life-threatening or fatal AEs that are definitely related to the intervention (defined in 9.3.3.2) from the time of informed consent signed to the end of visit 7 - must be entered in the CRFs within 24 hours of awareness and assessed by the site investigator.

All AEs from the time of informed consent signed to the end of visit 7 needs to be entered in the CRFs and assessed by the site investigator within 7 days of awareness. Participant self-reported AE (in daily eDiary) needs to be followed up and entered in REDCap (Research Electronic Data Capture) AE log, and assessed by site investigator.

9.3.6 SERIOUS ADVERSE EVENT REPORTING

All Serious Adverse Events (SAE) must be reported in the REDCap database by the site investigator within 24 hours of becoming aware of the SAE. The initial report should contain as much information as available.

At a minimum, the report must contain:

- Name of Site and Qualified Investigator,
- Participant Identification Code,
- Adverse Event Term,
- Study Drug Dose and Start/Stop Dates

Fatal or life-threatening unexpected adverse drug reactions must be reported to Health Canada within 7 days after awareness. All other serious unexpected adverse drug reactions must be reported within 15 days after awareness.

9.3.7 REPORTING EVENTS TO PARTICIPANTS

If cannabis-related SAEs occur, study participants will be notified based on recommendations from the DSMB and Steering Committee to the Principal Investigator.

9.3.8 REPORTING OF PREGNANCY

Pregnancy tests (detection of human chorionic gonadotropin) will be completed using blood samples collected to investigate cannabinoid blood levels. At visit 1, a urine pregnancy test will be added on top of the blood pregnancy test to exclude patients with positive result from enrollment. Eligibility confirmation will still depend on the visit 1 blood pregnancy test result. Any participant who becomes pregnant will be offered to counsel and will be withdrawn from the study (investigational product use terminated).

10 STATISTICAL CONSIDERATIONS

10.1 STATISTICAL HYPOTHESES

The primary endpoint (adverse events) will be analysed and presented descriptively with frequency counts and severity criteria. There are no pre specified statistical hypothesis.

10.2 SAMPLE SIZE DETERMINATION

The CAN-CHA trial is a tolerability study designed to identify a safe dose of a cannabis product in adolescents with chronic headache. Because of the within-participant study design, a sample size of 20 study participants should provide a reasonable characterization of the pattern of adverse event frequency and severity as dosage increases. To provide more generalizable data, we will recruit these 20 study participants across three chronic pain programs in Halifax, Vancouver, and Toronto.

10.3 POPULATIONS FOR ANALYSES

The following study populations are defined and will be analyzed as specified below.

Intention to treat population: We will complete an intent to treat analysis by which all participants who received at least one dose of the lowest dose of investigational product, whose dosing went off the track, lost to follow up, non-compliance, protocol deviation, and withdrawal.

Any study participant on this trial (registered) but never got study treatment will be mentioned, including the reason(s) of their exclusion from the trial.

10.3.1 SAFETY ANALYSES

System Organ Class (SOC) will present the severity, frequency, and relationship of AEs to study intervention will be presented by System Organ Class (SOC). The start date, stop date, severity, relationship, expectedness, outcome, and duration of each AE will be reported. Serious treatment-emergent AEs will be presented with descriptions of causality and management.

10.3.2 BASELINE DESCRIPTIVE STATISTICS

The primary statistical analysis will be a descriptive summary of the pattern of incidence and severity of adverse events across dosage levels. Conventional summary statistics will describe baseline characteristics and other outcomes (means, standard deviations, and medians, range and interquartile range (IQR) for numerical variables; counts and percentages for categorical variables). Summaries will be provided both overall and by dosage level. Medians, ranges and IQR, will be provided for the concentrations of CBD, THC and the primary metabolites at each sampling point. The ratio of parent compound concentration to metabolites will also be summarized to explore variability in cannabinoid metabolism. System organ class will present the severity, frequency, and relationship of treatment-emergent AEs to study intervention. The start date, stop date, severity, relationship, expectedness, outcome, and duration of each AE will be reported. Serious treatment-emergent AEs will be presented either in a table or a listing. The secondary outcomes, including pain, sleep impairment, depression, positive affect, anxiety and goal attainment scores, will be summarized at each timepoint; change from baseline will also be reported (both absolute and percentage change).

10.3.3 SUB-GROUP ANALYSES

We will perform subgroup analysis of primary and secondary endpoints based on sex and dose levels.

10.3.4 TABULATION OF INDIVIDUAL PARTICIPANT DATA

Individual participant data will be listed by measure and time point.

11 SUPPORTING DOCUMENTATION AND OPERATIONAL CONSIDERATIONS

11.1 REGULATORY, ETHICAL, AND STUDY OVERSIGHT CONSIDERATIONS

11.1.1 INFORMED CONSENT PROCESS

11.1.1.1 CONSENT/ASSENT AND OTHER INFORMATIONAL DOCUMENTS PROVIDED TO PARTICIPANTS

Consent forms describing the study intervention, study procedures, and risks are given to the participant, and written documentation of informed consent is required before starting intervention/administering study intervention.

11.1.1.2 CONSENT AND ASSENT PROCEDURES AND DOCUMENTATION

Informed consent/assent is a process that is initiated before the individual agrees to participate in the study and continues throughout the individual's study participation. Consent and assent forms will be Research Ethics Board (REB)-approved, and the participant will be asked to read and review the document. The site investigator will explain the medical part of the research study to the participant and answer any questions that may arise. A verbal explanation will be provided in terms of the participant's comprehension of the purposes, procedures, and potential risks of the study and their rights as research participants. Caregivers (if applicable) and/or participants will have the opportunity to carefully review the written consent and/or assent form(s) and ask questions before signing. The participants should have a chance to discuss the study with their family or surrogates or think about it before agreeing to participate. The caregiver and/or participant will sign the informed consent and/or assent document(s) before any procedures being done specifically for the study. Caregivers and/or participants must be informed that participation is voluntary and that they may withdraw from the study at any time without prejudice. A copy of the informed consent/assent document(s) will be given to the participants for their records. The informed consent/assent process will be conducted and documented in the source document (including the date). The rights and welfare of the participants will be protected by emphasizing to them that the quality of their medical care will not be adversely affected if they decline to participate in this study.

11.1.1.3 STUDY DISCONTINUATION AND CLOSURE

This study may be temporarily suspended or prematurely terminated if there is sufficient reasonable cause. Written notification, documenting the reason for study suspension or termination, will be provided by the suspending or terminating party to study participants, investigator, funding agency, and regulatory authorities. Suppose the study is prematurely terminated or suspended. In that case, the site Qualified investigator (QI) will promptly inform study participants, the REB, and the sponsor and will provide the reason(s) for the termination or suspension. Study participants will be contacted, as applicable, and be informed of changes to the study visit schedule.

Circumstances that may warrant termination or suspension include, but are not limited to:

- Determination of unexpected, significant, or unacceptable risk to participants
- Demonstration of efficacy that would warrant stopping
- Insufficient compliance to protocol requirements
- Data that are not sufficiently complete and/or evaluable
- Determination that the primary endpoint has been met
- Determination of futility

The study may resume once concerns about safety, protocol compliance, and data quality are addressed and satisfy the sponsor and/or REB.

11.1.2 CONFIDENTIALITY AND PRIVACY

Participant confidentiality and privacy is strictly held in trust by the participating investigators, their staff, and the sponsor(s) and their interventions. This confidentiality is extended to cover the testing of biological samples in addition to the clinical and genetic information relating to participants. Therefore, the study protocol, documentation, data, and all other information generated will be held in strict confidence. Furthermore, no information concerning the study of the data will be released to any unauthorized third party without the prior written approval of the sponsor.

All research activities will be conducted in as private a setting as possible.

The study monitor, other authorized representatives of the sponsor, representatives of the Research Ethics Board (REB), regulatory agencies may inspect all documents and records required to be maintained by the qualified investigators, including but not limited to medical records (office, clinic, or hospital) and pharmacy records for the participants in this study.

The study participant's contact information will be securely stored at each clinical site for internal use during the study. At the end of the study, all records will continue to be kept in a secure location for at least 15 years after study completion per Health Canada requirement, and as long as dictated by the reviewing REB, Institutional policies, or sponsor requirements.

Study participant research data, which is for statistical analysis and scientific reporting, will be transmitted to and stored at the Women and Children's Health Research Institute, University of Alberta. Edmonton. This will not include the participant's identifying information. Rather, individual participants and their research data will be determined by a unique study identification number. The study data entry and study management systems used by clinical sites will be secured and password protected. At the end of the study, all study data will be archived at University of Manitoba.

11.1.3 FUTURE USE OF STORED SPECIMENS AND DATA

Data collected for this study will be analyzed and stored at the Women and Children's Health Research Institute (WCHRI) at the University of Alberta, Edmonton. After the study is completed, the de-identified, archived data will be transmitted to and stored at the University of Manitoba for use by other researchers, including those outside the

study. Permission to transfer data from WCHRI to the University of Manitoba will be included in the informed consent.

11.1.4 KEY ROLES AND STUDY GOVERNANCE

Principal Investigator	Trial Manager
Dr. Lauren Kelly, PhD	Wenli Xie, MSc
Associate Professor, Departments of Pharmacology and Therapeutics, University of Manitoba	Clinical Trials Manager,<br>The Canadian Collaborative for Childhood Cannabinoid Therapeutics (C4T) & MICYRN

Expert Steering Committee Members	
Dr. Daniela Pohl, MD, PhD	Professor, Pediatric Neurologist<br>University of Ottawa
Dr. Richard Huntsman MD, FRCPC	Associate Professor, Pediatric Neurologist<br>University of Saskatchewan College of Medicine
Dr. Robert Balshaw, PhD	Statistician<br>University of Manitoba
Dr. Alexander Weil, MD FRCSC, FAANS, FACS	Pediatric Neurosurgeon<br>Université de Montréal
Trinity Lowthian	Youth partner with lived experience with chronic headache
Zahra Alidina	Youth partner with lived experience with chronic headache
Melila Chesick-Gordis	Youth partner with lived experience with chronic headache

11.1.5 SAFETY OVERSIGHT

The operations of the trial will be governed by the CAN-CHA steering committee under predefined terms of reference. Safety oversight will be under the direction of a Data and Safety Monitoring Board (DSMB) composed of Chair and voting members with expertise in pediatrics, clinical trials, pharmacology, particularly cannabis, and pain mitigation in children. Members of the DSMB are independent from the study conduct and free of conflict of interest. The DSMB will meet as indicated in DSMB charter to assess safety and efficacy data. At that time, each data element that the DSMB needs to assess will be clearly defined.

The primary responsibilities of the DSMB are as follows:

1. Review and evaluate the accumulated study data for participant safety.
2. Make recommendations to the Sponsor/Study Committee based on these reviews regarding the continuation, modification, or termination of the trial.
3. DSMB members must maintain strict confidentiality concerning all privileged trial results, and during all phases of DSMB review and deliberations.
4. No member of the DSMB should have a direct involvement with the conduct of the study. No member should have financial, proprietary, professional, or other interests that may affect impartial, independent decision-making by the DSMB.

The sponsor will notify the DSMB of all suspected unexpected serious adverse drug reactions (SUSARS) and all deaths at regular or ad hoc meetings of the DSMB.

All correspondence with the DSMB will be filed in the study records.

Additional data may be requested by the DSMB, and reports may be generated for review as deemed necessary and appropriate. The DSMB may receive data in aggregate and presented by the treatment arm. The DSMB may also be provided with expected and observed rates of the expected SAEs. As an outcome of each review/meeting, the DSMB will make a recommendation as to the advisability of proceeding with study interventions (as applicable), and to continue, modify, or terminate this trial.

11.1.6 CLINICAL MONITORING

Clinical site monitoring is conducted to ensure that the rights and well-being of trial participants are protected, that the reported trial data are accurate, complete, and verifiable, and that the conduct of the trial is in compliance with the currently approved protocol/amendment(s), with International Conference on Harmonisation Good Clinical Practice (ICH GCP), and with applicable regulatory requirement(s).

The Sponsor will be responsible for all monitoring activities. Any trial-related duty or function transferred to and assumed by a third party, including monitoring and auditing, will be specified in a clinical trial agreement and oversight provided by the Sponsor.

The monitoring plan for the trial will be documented prior to the activation of the study and include the following;

- Follow risk-based practices,
- Document the rationale for the chosen monitoring strategy,
- Reference the Sponsor's process that will be followed to address situations of non-compliance,
- Describe the monitoring responsibilities of all the parties involved, and
- Outline the data and processes to be monitored.

The site Investigator(s)/delegate(s) will allow direct access to source data/documents for the purposes of monitoring by the Sponsor, and inspection by local and regulatory authorities. It is important that the Sponsor, site Investigator and site personnel are available during monitoring visits and inspections, and that sufficient time is devoted to the process.

Monitoring procedures will be implemented beginning with the data entry system and reports of data checks that will be run on the database will be generated. Any missing data or data anomalies will be communicated to the site(s) for clarification/resolution.

Monitoring reports will be issued after each monitoring visit for review and follow up by the Sponsor, site Investigator, and appropriate management and personnel responsible for trial and site oversight.

11.1.7 QUALITY ASSURANCE AND QUALITY CONTROL

Quality control (QC) procedures will be implemented beginning with the data entry system and data QC checks that will be run on the database will be generated. Any missing data or data anomalies will be communicated to the site(s) for clarification/resolution.

Trial monitors will verify that the clinical trial is conducted, and data are generated and biological specimens are collected, documented (recorded), and reported in compliance with the protocol, International Conference on Harmonization Good Clinical Practice (ICH GCP), Health Canada Division 5 (Part C, Division 5 of the Food and Drug Regulations “Drugs for Clinical Trials Involving Human Subjects”), and applicable regulatory requirements.

11.1.8 DATA HANDLING AND RECORD KEEPING

Data will be deposited in a RedCap database and reviewed by the study monitor. Source documentation will be maintained by participating site study personnel in accordance with institutional policies so that the conduct of the trial and treatment of study participants can be verified by monitoring oversight.

Records and documents, including signed ICFs, pertaining to the conduct of this study must be retained by the site QIs for 15 years after study completion unless local regulations or institutional policies require a longer retention period. No records will be destroyed during the retention period without the written approval of the sponsor. No records may be transferred to another location or party without written notification to the sponsor.

11.1.8.1 DATA COLLECTION AND MANAGEMENT RESPONSIBILITIES

Data collection is the responsibility of the clinical trial staff at the site under the supervision of the site investigator. Site investigator is responsible for ensuring the accuracy, completeness, legibility, and timeliness of the data reported.

All source documents should be completed in a neat, legible manner to ensure accurate interpretation of data. Hardcopies of the study visit worksheets will be provided for use as source document worksheets for recording data for each participant enrolled in the study. Data recorded in the electronic case report form (eCRF) derived from source documents should be consistent with the data recorded on the source documents.

Clinical data (including adverse events (AEs), concomitant medications, and expected adverse reactions data) and clinical laboratory data will be entered into REDCap (Research Electronic Data Capture), a 21 CFR Part 11-compliant data capture system provided by the Women and Children’s Health Research Institute at the University of Alberta, Edmonton. The data system includes password protection and internal quality checks, such as automatic range checks, to identify data that appear inconsistent, incomplete, or inaccurate.

11.1.9 PROTOCOL DEVIATIONS

A protocol deviation is any noncompliance with the clinical trial protocol, International Conference on Harmonisation Good Clinical Practice (ICH GCP), or Manual of Procedures (MOP) requirements. The noncompliance may be either on the part of the

participant, the investigator, or the study site staff. As a result of deviations, corrective actions are to be developed by the site and implemented promptly. The site Qualified Investigators should ensure that no deviation from, or changes to the protocol will take place without prior agreement from the Sponsor and documented approval from the Research Ethics Board (REB), except where necessary to eliminate an immediate hazard(s) to the trial participants. All deviations from the protocol must be documented.

It is the responsibility of the site investigator to use continuous vigilance to identify and report deviations within a timely manner of identification of the protocol deviation. All deviations must be addressed in study source documents, reported to study PI. Protocol deviations must be sent to the reviewing Research Ethics Board (REB) per their policies. The site investigator is responsible for knowing and adhering to the reviewing REB requirements for protocol deviation reporting.

11.1.10 CONFLICT OF INTEREST POLICY

The independence of this study from any actual or perceived influence, such as by the pharmaceutical industry, is critical. Therefore, any actual conflict of interest of persons who have a role in the design, conduct, analysis, publication, or any aspect of this trial will be disclosed and managed. Furthermore, persons who have a perceived conflict of interest will be required to have such conflicts managed in a way that is appropriate to their participation in the design and conduct of this trial.

The Principal Investigator will be responsible for managing declared Conflicts of Interest.

11.2 ABBREVIATIONS

ADR	Adverse Drug Reaction
AE	Adverse Event
2-AG	2-Arachidonoyl-sn-Glycerol
BMI	Body Mass Index
CAN	Cannabis
CFR	Code of Federal Regulations
CHE	Cannabis Herbal Extract
CHI	The George & Fay Yee Centre for Healthcare Innovation
CHRIM	Children’s Hospital Research Institute of Manitoba
CM	Chronic Migraine
CMP	Clinical Monitoring Plan
CNS	Central Nervous System
CRF	Case Report Form
CTTH	Chronic Tension Type of Headache
THC	Tetrahydrocannabinol
TTH	Tension Type Headache
DCC	Data Coordinating Center
DSMB	Data Safety Monitoring Board
DS	Dravet Syndrome
ECG	Electrocardiogram

eCRF	Electronic Case Report Forms
FAAH	Fatty Acid Amide Hydrolase
FDA	Food and Drug Administration
GCP	Good Clinical Practice
GSA	Global Screening Array
GWAS	Genome-Wide Association Studies
HIPAA	Health Insurance Portability and Accountability Act
IB	Investigator’s Brochure
ICH	International Conference on Harmonisation
ITT	Intention-To-Treat
LGS	Lennox–Gastaut Syndrome
MCT	Medium Chain Triglyceride
MOH	Medication Overuse Headache
MOP	Manual of Procedures
NDPH	New Daily Persistent Headache
NCT	National Clinical Trial
NSAIDS	Non-steroidal Anti-inflammatory Drugs
NRS	Numeric Rating Scale
PROMIS	Patient Reported Outcome Measurement Information System
PPARs	Peroxisome Proliferator-Activated Receptor Gamma
PI	Principal Investigator
QA	Quality Assurance
QC	Quality Control
QI	Qualified Investigator
REDCap	Research Electronic Data Capture
RCTs	Randomized Control Trial
REB	Research Ethics Board
SAE	Serious Adverse Event
SAP	Statistical Analysis Plan
SOA	Schedule of Activities
SOC	System Organ Class
SOP	Standard Operating Procedure
THC	Tetrahydrocannabinol
TRPV1	Transient Receptor potential cation channel subfamily V 1
UP	Unanticipated Problem
US	United States

11.3 PROTOCOL AMENDMENT HISTORY

The table below is intended to capture changes of REB-approved versions of the protocol, including a description of the change and rationale.

**Version**	**Date**	**Description of Change**	**Brief Rationale**
2.0	19 Oct 2022	updated study IP: MPL-001	

3.0	05 Feb 2023	Term clarification and updates	To further clarify trial operation process
4.0	20 Mar 2023	Remove keeping samples for future studies part	Study samples will not be kept for future research
5.0	30 May 2023	Remove the question of number of headaches in daily eDiary and clarify dose-rounding method in IP dose calculation	Instead of collecting the duration of each headache event, this study will collect number of headache-free days per month via eDiary; participant's IP dose will be rounding off the nearest 1 mg CBD.
6.0	01 Mar 2024	Add the Cannabis Use Disorder Identification Test Short-Form questionnaire assessment on visit 1 and visit 7; delete duplicate and redundancy info in protocol	To collect cannabis usage data to inform future studies

12 REFERENCES

1. Wright, S., et al., *Cannabidiol (CBD) in dravet syndrome: A randomised, dose-ranging pharmacokinetics and safety trial (GWPCARE1).* Epilepsia, 2017. **58**: p. S56.
2. Wirrell, E., et al., *Cannabidiol (CBD) significantly reduces drop and total seizure frequency in lennox gastaut syndrome (LGS): Results of a dose ranging, multicenter, randomized, double blind, placebo controlled trial (GWPCARE3).* Annals of Neurology, 2017. **82**: p. S279-S280.
3. Thiele, E., et al., *Cannabidiol in patients with Lennox-Gastaut syndrome: Interim analysis of an open-label extension study.* Epilepsia, 2019.
4. Devinsky, O., et al., *Cannabidiol in patients with treatment-resistant epilepsy: an open-label interventional trial.* The Lancet. Neurology, 2016. **15**(3): p. 270-278.
5. Devinsky, O., et al., *Effect of Cannabidiol on Drop Seizures in the Lennox-Gastaut Syndrome.* N Engl J Med, 2018. **378**(20): p. 1888-1897.
6. Devinsky, O., et al., *Efficacy and safety of epidiolex (cannabidiol) in children and young adults with treatment-resistant epilepsy: Initial data from an expanded access program.* Epilepsy Currents, 2015. **15**: p. 532.

7. Devinsky, O., et al., *Long-term cannabidiol treatment in patients with Dravet syndrome: An open-label extension trial.* Epilepsia, 2019. **60**(2): p. 294-302.

8. Szaflarski, J.P., et al., *Long-term safety and treatment effects of cannabidiol in children and adults with treatment-resistant epilepsies: Expanded access program results.* Epilepsia, 2018. **59**(8): p. 1540-1548.

9. Devinsky, O., et al., *Trial of Cannabidiol for Drug-Resistant Seizures in the Dravet Syndrome.* The New England journal of medicine, 2017. **376**(21): p. 2011-2020.

10. Russo, E., *Hemp for headache: an in-depth historical and scientific review of cannabis in migraine treatment.* Journal of Cannabis Therapeutics, 2001. **1**(2): p. 21-92.

11. Seshia, S.S., et al., *Chronic daily headache in children and adolescents: a multi-faceted syndrome.* Can J Neurol Sci, 2010. **37**(6): p. 769-78.

12. Stovner, L., et al., *The global burden of headache: a documentation of headache prevalence and disability worldwide.* Cephalalgia, 2007. **27**(3): p. 193-210.

13. Castillo, J., et al., *Kaplan Award 1998. Epidemiology of chronic daily headache in the general population.* Headache, 1999. **39**(3): p. 190-6.

14. Lantéri-Minet, M., et al., *Prevalence and description of chronic daily headache in the general population in France.* Pain, 2003. **102**(1-2): p. 143-9.

15. Grande, R.B., et al., *Prevalence of primary chronic headache in a population-based sample of 30- to 44-year-old persons. The Akershus study of chronic headache.* Neuroepidemiology, 2008. **30**(2): p. 76-83.

16. Ahmed, F., *Headache disorders: differentiating and managing the common subtypes.* British journal of pain, 2012. **6**(3): p. 124-132.

17. Bellini, B., et al., *Headache and comorbidity in children and adolescents.* The journal of headache and pain, 2013. **14**(1): p. 79-79.

18. Ailani, J., *Chronic tension-type headache.* Curr Pain Headache Rep, 2009. **13**(6): p. 479-83.

19. Kristoffersen, E.S. and C. Lundqvist, *Medication-overuse headache: epidemiology, diagnosis and treatment.* Therapeutic advances in drug safety, 2014. **5**(2): p. 87-99.

20. Tepper, D., *New Daily Persistent Headache.* Headache, 2016. **56**(7): p. 1249-50.

21. Pardutz, A. and J. Schoenen, *NSAIDs in the Acute Treatment of Migraine: A Review of Clinical and Experimental Data.* Pharmaceuticals (Basel, Switzerland), 2010. **3**(6): p. 1966-1987.

22. Ravishankar, K., et al., *Guidelines on the diagnosis and the current management of headache and related disorders.* Annals of Indian Academy of Neurology, 2011. **14**(Suppl 1): p. S40-S59.

23. Kacperski, J., et al., *The optimal management of headaches in children and adolescents.* Therapeutic advances in neurological disorders, 2016. **9**(1): p. 53-68.

24. Seshia, S.S. and R.J. Huntsman, *An Adolescent with Chronic Daily Headache*, in *Headache in Children and Adolescents*. 2016, Springer. p. 85-93.

25. Lochte, B.C., et al., *The Use of Cannabis for Headache Disorders.* Cannabis and cannabinoid research, 2017. **2**(1): p. 61-71.

26. Cuttler, C., et al., *Short- and Long-Term Effects of Cannabis on Headache and Migraine.* J Pain, 2019.

27. Steiner, T.J., *Lifting the burden: The global campaign against headache.* Lancet Neurol, 2004. **3**(4): p. 204-5.

28. Stewart, W.F., R.B. Lipton, and J. Liberman, *Variation in migraine prevalence by race.* Neurology, 1996. **47**(1): p. 52-9.

29. Lipton, R.B., S.D. Silberstein, and W.F. Stewart, *An update on the epidemiology of migraine.* Headache, 1994. **34**(6): p. 319-28.

30. Stewart, W.F., et al., *Age- and sex-specific incidence rates of migraine with and without visual aura.* Am J Epidemiol, 1991. **134**(10): p. 1111-20.

31. Abu-Arafeh, I., et al., *Prevalence of headache and migraine in children and adolescents: a systematic review of population-based studies.* Dev Med Child Neurol, 2010. **52**(12): p. 1088-97.

32. Wang, S.J., et al., *Chronic daily headache in adolescents: prevalence, impact, and medication overuse.* Neurology, 2006. **66**(2): p. 193-7.

33. Bigal, M.E. and M.A. Arruda, *Migraine in the pediatric population--evolving concepts.* Headache, 2010. **50**(7): p. 1130-43.

34. Dodick, D. and F. Freitag, *Evidence-based understanding of medication-overuse headache: clinical implications.* Headache, 2006. **46 Suppl 4**: p. S202-11.

35. Arruda, M.A. and M.E. Bigal, *Behavioral and emotional symptoms and primary headaches in children: a population-based study.* Cephalalgia, 2012. **32**(15): p. 1093-100.

36. Galli, F., et al., *Headache and recurrent abdominal pain: a controlled study by the means of the Child Behaviour Checklist (CBCL).* Cephalalgia, 2007. **27**(3): p. 211-9.

37. Bruijn, J., et al., *Psychopathology in children and adolescents with migraine in clinical studies: a systematic review.* Pediatrics, 2010. **126**(2): p. 323-32.

38. Dosi, C., et al., *Comorbidities of sleep disorders in childhood and adolescence: focus on migraine.* Nature and science of sleep, 2013. **5**: p. 77-85.

39. Owens, J.A. and M. Witmans, *Sleep problems.* Curr Probl Pediatr Adolesc Health Care, 2004. **34**(4): p. 154-79.

40. Roth-Isigkeit, A., et al., *Pain among children and adolescents: restrictions in daily living and triggering factors.* Pediatrics, 2005. **115**(2): p. e152-62.

41. Gilman, D.K., et al., *Primary headache and sleep disturbances in adolescents.* Headache, 2007. **47**(8): p. 1189-94.

42. Sieberg, C.B., et al., *Psychological interventions for headache in children and adolescents.* Can J Neurol Sci, 2012. **39**(1): p. 26-34.

43. Moreno, M.A., F. Furtner, and F.P. Rivara, *Treating Headaches in Children and Adolescents.* JAMA Pediatrics, 2013. **167**(3): p. 308-308.

44. Lewis, D., et al., *Practice parameter: pharmacological treatment of migraine headache in children and adolescents: report of the American Academy of Neurology Quality Standards Subcommittee and the Practice Committee of the Child Neurology Society.* Neurology, 2004. **63**(12): p. 2215-24.

45. Hershey, A.D., et al., *Effectiveness of amitriptyline in the prophylactic management of childhood headaches.* Headache, 2000. **40**(7): p. 539-49.

46. Gillman, P.K., *Tricyclic antidepressant pharmacology and therapeutic drug interactions updated.* British journal of pharmacology, 2007. **151**(6): p. 737-748.

47. Ahmed, A.I., P. van Mierlo, and P. Jansen, *Sleep disorders, nightmares, depression and anxiety in an elderly patient treated with low-dose metoprolol.* Gen Hosp Psychiatry, 2010. **32**(6): p. 646.e5-7.

48. Figueroa, J.J., J.R. Basford, and P.A. Low, *Preventing and treating orthostatic hypotension: As easy as A, B, C.* Cleveland Clinic journal of medicine, 2010. **77**(5): p. 298-306.

49. Hörl, W.H., *Nonsteroidal Anti-Inflammatory Drugs and the Kidney.* Pharmaceuticals (Basel, Switzerland), 2010. **3**(7): p. 2291-2321.

50. McGovern, M.C., J.F. Glasgow, and M.C. Stewart, *Lesson of the week: Reye's syndrome and aspirin: lest we forget.* BMJ (Clinical research ed.), 2001. **322**(7302): p. 1591-1592.

51. Glasgow, J.F., *Reye's syndrome: the case for a causal link with aspirin.* Drug Saf, 2006. **29**(12): p. 1111-21.

52. Hare, H.A., *Clinical and physiological notes on the action of Cannabis indica.* Therap Gaz, 1887. **2**: p. 225-228.

53. Gonçalves, J., et al., *Cannabis and Its Secondary Metabolites: Their Use as Therapeutic Drugs, Toxicological Aspects, and Analytical Determination.* Medicines (Basel, Switzerland), 2019. **6**(1): p. 31.

54. Morales, P., et al., *Allosteric Modulators of the CB(1) Cannabinoid Receptor: A Structural Update Review.* Cannabis Cannabinoid Res, 2016. **1**(1): p. 22-30.

55. Pertwee, R.G., *Pharmacology of cannabinoid receptor ligands.* Curr Med Chem, 1999. **6**(8): p. 635-64.

56. el-Mallakh, R.S., *Marijuana and migraine.* Headache, 1987. **27**(8): p. 442-3.

57. Grinspoon, L.G., L. Grinspoon, and J.B. Bakalar, *Marihuana, the forbidden medicine*. 1997: Yale University Press.

58. Rhyne, D.N., et al., *Effects of Medical Marijuana on Migraine Headache Frequency in an Adult Population.* Pharmacotherapy, 2016. **36**(5): p. 505-10.

59. Mikuriya, T., *Chronic migraine headache: Five cases successfully treated with marinol and/or illicit cannabis. 1991*.

60. Nunberg, H., et al., *An analysis of applicants presenting to a medical marijuana specialty practice in California.* Journal of drug policy analysis, 2011. **4**(1).

61. Pini, L.A., et al., *Nabilone for the treatment of medication overuse headache: results of a preliminary double-blind, active-controlled, randomized trial.* J Headache Pain, 2012. **13**(8): p. 677-84.

62. Chesher, G.B., et al., *The effect of cannabinoids on intestinal motility and their antinociceptive effect in mice.* Br J Pharmacol, 1973. **49**(4): p. 588-94.

63. Sofia, R.D., H.B. Vassar, and L.C. Knobloch, *Comparative analgesic activity of various naturally occurring cannabinoids in mice and rats.* Psychopharmacologia, 1975. **40**(4): p. 285-95.

64. Bensemana, D. and A.L. Gascon, *Relationship between analgesia and turnover of brain biogenic amines.* Can J Physiol Pharmacol, 1978. **56**(5): p. 721-30.

65. Jaggar, S.I., et al., *The anti-hyperalgesic actions of the cannabinoid anandamide and the putative CB2 receptor agonist palmitoylethanolamide in visceral and somatic inflammatory pain.* Pain, 1998. **76**(1-2): p. 189-99.

66. Clayton, N., et al., *CB1 and CB2 cannabinoid receptors are implicated in inflammatory pain.* Pain, 2002. **96**(3): p. 253-60.

67. Elmes, S.J., et al., *Cannabinoid CB2 receptor activation inhibits mechanically evoked responses of wide dynamic range dorsal horn neurons in naïve rats and in rat models of inflammatory and neuropathic pain.* Eur J Neurosci, 2004. **20**(9): p. 2311-20.

68. Cheng, Y. and S.A. Hitchcock, *Targeting cannabinoid agonists for inflammatory and neuropathic pain.* Expert Opin Investig Drugs, 2007. **16**(7): p. 951-65.

69. Rice, A.S., W.P. Farquhar-Smith, and I. Nagy, *Endocannabinoids and pain: spinal and peripheral analgesia in inflammation and neuropathy.* Prostaglandins Leukot Essent Fatty Acids, 2002. **66**(2-3): p. 243-56.

70. Johanek, L.M. and D.A. Simone, *Activation of peripheral cannabinoid receptors attenuates cutaneous hyperalgesia produced by a heat injury.* Pain, 2004. **109**(3): p. 432-42.

71. Papanastassiou, A.M., H.L. Fields, and I.D. Meng, *Local application of the cannabinoid receptor agonist, WIN 55,212-2, to spinal trigeminal nucleus caudalis differentially affects nociceptive and non-nociceptive neurons.* Pain, 2004. **107**(3): p. 267-75.

72. Scott, D.A., C.E. Wright, and J.A. Angus, *Evidence that CB-1 and CB-2 cannabinoid receptors mediate antinociception in neuropathic pain in the rat.* Pain, 2004. **109**(1-2): p. 124-31.

73. Jeske, N.A., et al., *Cannabinoid WIN 55,212-2 regulates TRPV1 phosphorylation in sensory neurons.* J Biol Chem, 2006. **281**(43): p. 32879-90.

74. Patwardhan, A.M., et al., *The cannabinoid WIN 55,212-2 inhibits transient receptor potential vanilloid 1 (TRPV1) and evokes peripheral antihyperalgesia via calcineurin.* Proc Natl Acad Sci U S A, 2006. **103**(30): p. 11393-8.

75. Hasanein, P. and M. Teimuri Far, *Effects of URB597 as an inhibitor of fatty acid amide hydrolase on WIN55, 212-2-induced learning and memory deficits in rats.* Pharmacol Biochem Behav, 2015. **131**: p. 130-5.

76. Ibrahim, M.M., et al., *CB2 cannabinoid receptor mediation of antinociception.* Pain, 2006. **122**(1-2): p. 36-42.

77. Rácz, I., et al., *CB1 receptors modulate affective behaviour induced by neuropathic pain.* Brain Res Bull, 2015. **114**: p. 42-8.

78. Romero-Sandoval, A., N. Nutile-McMenemy, and J.A. DeLeo, *Spinal microglial and perivascular cell cannabinoid receptor type 2 activation reduces behavioral hypersensitivity without tolerance after peripheral nerve injury.* Anesthesiology, 2008. **108**(4): p. 722-34.

79. Correa, F., et al., *A role for CB2 receptors in anandamide signalling pathways involved in the regulation of IL-12 and IL-23 in microglial cells.* Biochem Pharmacol, 2009. **77**(1): p. 86-100.

80. Beltramo, M., et al., *CB2 receptor-mediated antihyperalgesia: possible direct involvement of neural mechanisms.* Eur J Neurosci, 2006. **23**(6): p. 1530-8.

81. Shang, Y. and Y. Tang, *The central cannabinoid receptor type-2 (CB2) and chronic pain.* Int J Neurosci, 2017. **127**(9): p. 812-823.

82. Xi, Z.X., et al., *Brain cannabinoid CB_2 receptors modulate cocaine's actions in mice.* Nat Neurosci, 2011. **14**(9): p. 1160-6.

83. Zhang, H.Y., et al., *Cannabinoid CB2 receptors modulate midbrain dopamine neuronal activity and dopamine-related behavior in mice.* 2014. **111**(46): p. E5007-15.

84. Zhang, H.Y., et al., *Expression of functional cannabinoid CB(2) receptor in VTA dopamine neurons in rats.* Addict Biol, 2017. **22**(3): p. 752-765.

85. Navratilova, E., et al., *Pain relief produces negative reinforcement through activation of mesolimbic reward-valuation circuitry.* Proc Natl Acad Sci U S A, 2012. **109**(50): p. 20709-13.

86. Navratilova, E., C.W. Atcherley, and F. Porreca, *Brain Circuits Encoding Reward from Pain Relief.* Trends Neurosci, 2015. **38**(11): p. 741-750.

87. Richardson, J.D., S. Kilo, and K.M. Hargreaves, *Cannabinoids reduce hyperalgesia and inflammation via interaction with peripheral CB1 receptors.* Pain, 1998. **75**(1): p. 111-9.

88. Costa, B., et al., *Oral anti-inflammatory activity of cannabidiol, a non-psychoactive constituent of cannabis, in acute carrageenan-induced*

inflammation in the rat paw. Naunyn Schmiedebergs Arch Pharmacol, 2004. **369**(3): p. 294-9.

89. Jayamanne, A., et al., *Actions of the FAAH inhibitor URB597 in neuropathic and inflammatory chronic pain models.* Br J Pharmacol, 2006. **147**(3): p. 281-8.
90. Desroches, J., et al., *Modulation of the anti-nociceptive effects of 2-arachidonoyl glycerol by peripherally administered FAAH and MGL inhibitors in a neuropathic pain model.* Br J Pharmacol, 2008. **155**(6): p. 913-24.
91. Kinsey, S.G., et al., *The CB2 cannabinoid receptor-selective agonist O-3223 reduces pain and inflammation without apparent cannabinoid behavioral effects.* Neuropharmacology, 2011. **60**(2-3): p. 244-51.
92. Long, J.Z., et al., *Dual blockade of FAAH and MAGL identifies behavioral processes regulated by endocannabinoid crosstalk in vivo.* Proc Natl Acad Sci U S A, 2009. **106**(48): p. 20270-5.
93. Ahn, K., et al., *Discovery and characterization of a highly selective FAAH inhibitor that reduces inflammatory pain.* Chem Biol, 2009. **16**(4): p. 411-20.
94. Peciña, M., et al., *FAAH selectively influences placebo effects.* Mol Psychiatry, 2014. **19**(3): p. 385-91.
95. Comelli, F., et al., *The inhibition of monoacylglycerol lipase by URB602 showed an anti-inflammatory and anti-nociceptive effect in a murine model of acute inflammation.* Br J Pharmacol, 2007. **152**(5): p. 787-94.
96. Guindon, J., et al., *Peripheral antinociceptive effects of inhibitors of monoacylglycerol lipase in a rat model of inflammatory pain.* Br J Pharmacol, 2011. **163**(7): p. 1464-78.
97. Schlosburg, J.E., et al., *Chronic monoacylglycerol lipase blockade causes functional antagonism of the endocannabinoid system.* Nat Neurosci, 2010. **13**(9): p. 1113-9.
98. Suardíaz, M., et al., *Analgesic properties of oleoylethanolamide (OEA) in visceral and inflammatory pain.* Pain, 2007. **133**(1-3): p. 99-110.
99. Luongo, L., et al., *Palmitoylethanolamide reduces formalin-induced neuropathic-like behaviour through spinal glial/microglial phenotypical changes in mice.* CNS Neurol Disord Drug Targets, 2013. **12**(1): p. 45-54.
100. O'Sullivan, S.E., *Cannabinoids go nuclear: evidence for activation of peroxisome proliferator-activated receptors.* Br J Pharmacol, 2007. **152**(5): p. 576-82.
101. Costa, B., et al., *The endogenous fatty acid amide, palmitoylethanolamide, has anti-allodynic and anti-hyperalgesic effects in a murine model of neuropathic pain: involvement of CB(1), TRPV1 and PPARgamma receptors and neurotrophic factors.* Pain, 2008. **139**(3): p. 541-50.
102. Bettoni, I., et al., *Non-neuronal cell modulation relieves neuropathic pain: efficacy of the endogenous lipid palmitoylethanolamide.* CNS Neurol Disord Drug Targets, 2013. **12**(1): p. 34-44.

103. Mecha, M., et al., *Cannabidiol provides long-lasting protection against the deleterious effects of inflammation in a viral model of multiple sclerosis: a role for A2A receptors.* Neurobiol Dis, 2013. **59**: p. 141-50.

104. Johnson, J.R., et al., *Multicenter, double-blind, randomized, placebo-controlled, parallel-group study of the efficacy, safety, and tolerability of THC:CBD extract and THC extract in patients with intractable cancer-related pain.* J Pain Symptom Manage, 2010. **39**(2): p. 167-79.

105. Chagas, M.H., et al., *Effects of cannabidiol in the treatment of patients with Parkinson's disease: an exploratory double-blind trial.* J Psychopharmacol, 2014. **28**(11): p. 1088-98.

106. Zou, S. and U. Kumar, *Cannabinoid Receptors and the Endocannabinoid System: Signaling and Function in the Central Nervous System.* International journal of molecular sciences, 2018. **19**(3): p. 833.

107. Lötsch, J., I. Weyer-Menkhoff, and I. Tegeder, *Current evidence of cannabinoid-based analgesia obtained in preclinical and human experimental settings.* Eur J Pain, 2018. **22**(3): p. 471-484.

108. Kogan, N.M. and R. Mechoulam, *Cannabinoids in health and disease.* Dialogues in clinical neuroscience, 2007. **9**(4): p. 413-430.

109. Pacher, P., S. Bátkai, and G. Kunos, *The endocannabinoid system as an emerging target of pharmacotherapy.* Pharmacological reviews, 2006. **58**(3): p. 389-462.

110. McGuire, P., et al., *Cannabidiol (CBD) as an Adjunctive Therapy in Schizophrenia: A Multicenter Randomized Controlled Trial.* Am J Psychiatry, 2018. **175**(3): p. 225-231.

111. Naftali, T., et al., *Low-Dose Cannabidiol Is Safe but Not Effective in the Treatment for Crohn's Disease, a Randomized Controlled Trial.* Dig Dis Sci, 2017. **62**(6): p. 1615-1620.

112. Thiele, E.A., et al., *Cannabidiol in patients with seizures associated with Lennox-Gastaut syndrome (GWPCARE4): a randomised, double-blind, placebo-controlled phase 3 trial.* Lancet, 2018. **391**(10125): p. 1085-1096.

113. Devinsky, O., et al., *Randomized, dose-ranging safety trial of cannabidiol in Dravet syndrome.* Neurology, 2018. **90**(14): p. e1204-e1211.

114. Devinsky, O., et al., *Trial of Cannabidiol for Drug-Resistant Seizures in the Dravet Syndrome.* N Engl J Med, 2017. **376**(21): p. 2011-2020.

115. Lazaridis, D., et al., *Treatment of Seizures Associated with Lennox-Gastaut and Dravet Syndromes: A Focus on Cannabidiol Oral Solution.* P & T : a peer-reviewed journal for formulary management, 2019. **44**(5): p. 255-266.

116. Reithmeier, D., et al., *The protocol for the Cannabidiol in children with refractory epileptic encephalopathy (CARE-E) study: a phase 1 dosage escalation study.* 2018. **18**(1): p. 221.

117. Huntsman, R.J., et al., *Dosage Related Efficacy and Tolerability of Cannabidiol in Children With Treatment-Resistant Epileptic Encephalopathy: Preliminary Results of the CARE-E Study.* Front Neurol, 2019. **10**: p. 716.

118. Patel, A., et al., *A dose ranging safety and pharmacokinetic study of cannabidol (CBD) in children with Dravet syndrome (GWPCARE1).* Neurology, 2017. **88**(16).

119. Lochte, B.C., et al., *The Use of Cannabis for Headache Disorders.* Cannabis Cannabinoid Res, 2017. **2**(1): p. 61-71.

120. Almog, S., et al., *The pharmacokinetics, efficacy, and safety of a novel selective-dose cannabis inhaler in patients with chronic pain: A randomized, double-blinded, placebo-controlled trial.* Eur J Pain, 2020. **24**(8): p. 1505-1516.

121. *The International Classification of Headache Disorders, 3rd edition (beta version).* Cephalalgia, 2013. **33**(9): p. 629-808.

122. Heron, K.E., et al., *Using Mobile-Technology-Based Ecological Momentary Assessment (EMA) Methods With Youth: A Systematic Review and Recommendations.* J Pediatr Psychol, 2017. **42**(10): p. 1087-1107.

123. Krebs, E.E., T.S. Carey, and M. Weinberger, *Accuracy of the pain numeric rating scale as a screening test in primary care.* Journal of general internal medicine, 2007. **22**(10): p. 1453-1458.

124. Birnie, K.A., P.J. McGrath, and C.T. Chambers, *When does pain matter? Acknowledging the subjectivity of clinical significance.* Pain, 2012. **153**(12): p. 2311-2314.

125. Cella, D., et al., *The Patient-Reported Outcomes Measurement Information System (PROMIS) developed and tested its first wave of adult self-reported health outcome item banks: 2005-2008.* J Clin Epidemiol, 2010. **63**(11): p. 1179-94.

126. Yu, L., et al., *Development of short forms from the PROMIS™ sleep disturbance and Sleep-Related Impairment item banks.* Behavioral sleep medicine, 2011. **10**(1): p. 6-24.

127. Irwin, D.E., et al., *An item response analysis of the pediatric PROMIS anxiety and depressive symptoms scales.* Quality of life research : an international journal of quality of life aspects of treatment, care and rehabilitation, 2010. **19**(4): p. 595-607.

128. Varni, J.W., et al., *Psychometric properties of the PROMIS ® pediatric scales: precision, stability, and comparison of different scoring and administration options.* Quality of life research : an international journal of quality of life aspects of treatment, care and rehabilitation, 2014. **23**(4): p. 1233-1243.

129. Varni, J.W., et al., *PROMIS Pediatric Pain Interference Scale: an item response theory analysis of the pediatric pain item bank.* J Pain, 2010. **11**(11): p. 1109-19.

130. Gordon, J.E., C. Powell, and K. Rockwood, *Goal attainment scaling as a measure of clinically important change in nursing-home patients.* Age Ageing, 1999. **28**(3): p. 275-81.

131. Forrest, C.B., et al., *Development and Evaluation of the PROMIS(®) Pediatric Positive Affect Item Bank, Child-Report and Parent-Proxy Editions.* Journal of happiness studies, 2018. **19**(3): p. 699-718.

132. Varni, J.W., et al., *The PedsQL Family Impact Module: preliminary reliability and validity.* Health and quality of life outcomes, 2004. **2**: p. 55-55.

133. Witte, J.S., *Genome-wide association studies and beyond.* Annual review of public health, 2010. **31**: p. 9-20.

134. Chang, C.C., et al., *Second-generation PLINK: rising to the challenge of larger and richer datasets.* GigaScience, 2015. **4**: p. 7-7.

135. Li, Y., et al., *Genotype imputation.* Annual review of genomics and human genetics, 2009. **10**: p. 387-406.

136. Schultz, N. R. et al. *Evaluation of the psychometric properties of the cannabis use disorders identification test - revised among college students.* Addictive behaviors. 2019. [Online] 9511–15.

137. Coelho, S. G. et al. *Screening for cannabis use disorder among young adults: Sensitivity, specificity, and item-level performance of the Cannabis Use Disorders Identification Test - Revised.* Addictive behaviors. 2024. [Online] 148.

138. Adamson, S. J. et al. *An improved brief measure of cannabis misuse: the Cannabis Use Disorders Identification Test-Revised (CUDIT-R).* Drug and alcohol dependence. 2010. [Online] 110 (1–2), 137–143.

139. Bonn-Miller, M. O. et al. *Preliminary Development of a Brief Cannabis Use Disorder Screening Tool: The Cannabis Use Disorder Identification Test Short-Form.* Cannabis and cannabinoid research. 2016. [Online] 1 (1), 252–261.
