# Supplementary material for: A multi-centre, tolerability study of a cannabidiol-enriched Cannabis Herbal Extract for chronic headaches in adolescents: The CAN-CHA protocol
Source: PLoS One. 2024 Sep 20;19(9):e0290185. doi: 10.1371/journal.pone.0290185 (PMC11414974; doi:10.1371/journal.pone.0290185)
Supplement: S2 Data — (PDF) [file pone.0290185.s003.pdf]

Cannabinoid and metabolite assessment

An LC-MS/MS method will be used to quantify the plasma concentration of THC, CBD, 11-OH-THC, 7-OH-CBD, and Cannabichromene. Stock solutions of cannabinoids and their respective stable isotope-labeled internal standards (Cerilliant Corp., Round Rock, TX) will be prepared in methanol at 1 mg/mL and stored at -20 °C until use. Stock solutions will be serial diluted using blank human plasma to develop calibration curves. Low, medium, and high-quality control (QC) samples will determine acceptance of the analytical run. QC and calibration samples will be prepared fresh each day samples are analyzed. Calibration curves will be constructed from a plot of the peak area ratios of the cannabinoid and its respective internal standard against the nominal concentration. The linearity of the calibration curves will be determined with a linear least-squares regression analysis using 1/X as a weighting factor. Using FDA bioanalytical guidance, all calibration curves will be linear (r2 >0.99) and intra- and inter-day precision and accuracy will be within 15%, except at the limit of quantification which will need to be within 20%. For all QC samples for a given run calculated concentrations will be within 15% of their nominal values. The plasma sample extraction will involve addition of internal standard working solution (10 µL) followed by protein precipitation with cold acetonitrile and removal of lipids using Agilent Captiva EMR-Lipid well plates. Supernatant will be transferred to borosilicate tubes and dried under filtered air for 30 minutes at 37°C. Samples will be reconstituted with 200 mL mobile phase and 100 µL transferred into HPLC inserts, 5 µL will be further injected onto an Agilent Phenyl-hexyl small-bore (2.1 × 12.5 mm, 5 µm) column and guard column maintaining the column temperature at 30°C to separate cannabinoids. Mobile phase A (water containing 0.1 mM ammonium formate) and B (methanol containing 0.1 mM ammonium formate) will separate the cannabinoids using a 10 minute gradient method with a flow rate of 250 L/min. Positive ion

mode electrospray ionization (ESI), with an ion spray voltage of 5500 V, will be used to conduct multiple reaction monitoring (MRM) for quantification of CBD (quantifier ion m/z 315.1>193.2; qualifier ion m/z 315.1>259.2), THC (quantifier ion m/z 315.1>193.1; qualifier ion m/z 315.1>259.2), 11-OH-THC (quantifier ion m/z 331.1>193.1; qualifier ion m/z 331.1>201.0), CBC (quantifier ion m/z 315.1>193.2; qualifier ion m/z 315.1>259.2), CBD-d3 (318.2>196.1), THC-d3 (318.2>196.1), 11-OH-THC-d3 (334.2>196.1), and CBC-d9 (324.3>202.2) using a ABSciex 6500 QTRAP mass spectrometer (ABSciex, Concord, ON, Canada) and MultiQuant 3.0.1 Software. The temperature, gas source 1, gas source 2, curtain gas, entrance potential, and collision activated dissociation gas were set to 600°C, 70, 60, 50, 10 V, and 10, respectively. The experienced team at the Cannabis Research Initiative of Saskatchewan, University of Saskatchewan will conduct all the analyses of the samples collected from all the three study sites. The samples will be analyzed in the three batches starting within the three months of the collections. The stability analysis of cannabinoids stored at -80°C for three months indicates excellent stability as described previously [20].
